# Supplementary material for: Change in time spent visiting and experiences of green space following restrictions on movement during the COVID-19 pandemic: a nationally representative cross-sectional study of UK adults
Source: BMJ Open. 2021 Mar 8;11(3):e044067. doi: 10.1136/bmjopen-2020-044067 (PMC7942249; doi:10.1136/bmjopen-2020-044067)
Supplement: Supplementary data [file bmjopen-2020-044067supp001.pdf]

Supplementary Materials

Supplementary Table 1: Category mergers of the response categories (ethnicity, age, social grade, sex and dog ownership).

	**Original categories (Unweighted N)**	**New categories (Unweighted N)**
**Ethnicity**	White British (1659)	White (1745)
	Any other white background (86)	
	White and Black Caribbean (6)	BAME (87)
	White and Black African (4)	
	White and Asian (8)	
	Any other mixed background (8)	
	Indian (17)	
	Pakistani (5)	
	Bangladeshi (4)	
	Any other Asian background (3)	
	Black Caribbean (4)	
	Black African (3)	
	Any other black background (3)	
	Chinese (10)	
	Other ethnic group (12)	
	Prefer not to say (19)	Missing (420)
	Not answered (401)	
**Age**	18-24 (165)	18-24 (165)
	25-34 (316)	25-64 (1497)
	35-44 (396)	
	45-54 (398)	
	55-64 (387)	
	65+ (590)	65+ (590)
**Social grade**	ABC1 (1419)	
	C2DE (833)	
**Sex**	Male (1018)	
	Female (1234)	
**Dog ownership**	Yes (616)	
	No (1636)	

Supplementary Table 2: Multinomial logistic regression models of being in each outcome group for change in time visiting green space (increase or decrease in visits to green space since movement restrictions were enforced compared to before), results are reported as Risk Ratios (RR) with 95% Lower Confidence Limits (LCL) and Upper Confidence Limits (UCL); **p<0.05**.

		RR of Decreased visits to "Same" and 95% CIs			RR of Increased to "Same" and 95% CIs		
		RR	LCL	UCL	RR	LCL	UCL
	**Intercept**	3.11	2.48	3.90	0.96	0.71	1.30
Sex	**Female *(Ref=Male)***	**1.34**	**1.07**	**1.69**	1.19	0.85	1.66
Social Grade	**C2DE *(Ref=ABC1)***	**0.70**	**0.56**	**0.88**	**0.40**	**0.28**	**0.56**
Age group	**18-24 *(Ref=25-64)***	0.91	0.56	1.47	1.50	0.82	2.73
	**65+ *(Ref=25-64)***	1.13	0.87	1.46	**0.64**	**0.43**	**0.97**
Ethnicity	**BAME *(Ref=White)***	0.62	0.38	1.02	0.83	0.42	1.62
Dog Ownership	**Yes *(Ref=No)***	**0.69**	**0.53**	**0.88**	**0.56**	**0.38**	**0.82**

Supplementary Table 3: Multinomial logistic regression models of being in each outcome group for levels of agreement that green space benefits their mental health more now (since movement restrictions were enforced compared to before), results are reported as Risk Ratios (RR) with 95% Lower Confidence Limits (LCL) and Upper Confidence Limits (UCL); **p<0.05**.

		RR of Agree to "Neither agree or disagree" and 95% CIs			RR of Disagree to "Neither agree or disagree" and 95% CIs		
		RR	LCL	UCL	RR	LCL	UCL
	**Intercept**	2.94	2.17	3.99	0.43	0.27	0.70
Sex	**Female *(Ref=Male)***	**1.72**	**1.24**	**2.39**	1.29	0.76	2.18
Social Grade	**C2DE *(Ref=ABC1)***	**0.67**	**0.48**	**0.93**	0.88	0.52	1.49
Age group	**18-24 *(Ref=25-64)***	0.87	0.40	1.89	**2.71**	**1.03**	**7.12**
	**65+ *(Ref=25-64)***	**0.47**	**0.33**	**0.67**	**0.37**	**0.19**	**0.73**
Ethnicity	**BAME *(Ref=White)***	1.20	0.54	2.69	0.21	0.03	1.43
Dog Ownership	**Yes *(Ref=No)***	0.72	0.51	1.02	1.02	0.59	1.76

Supplementary Table 4: Multinomial logistic regression models of being in each outcome group for levels of agreement that respondents missed seeing/talking to people in green space more since movement restrictions were enforced compared to before, results are reported as Risk Ratios (RR) with 95% Lower Confidence Limits (LCL) and Upper Confidence Limits (UCL); **p<0.05**.

		RR of Agree to "Neither agree or disagree" and 95% CIs			RR of Disagree to "Neither agree or disagree" and 95% CIs		
		RR	LCL	UCL	RR	LCL	UCL
	**Intercept**	1.47	1.08	1.99	0.89	0.62	1.27
Sex	**Female *(Ref=Male)***	**1.53**	**1.11**	**2.13**	0.76	0.50	1.14
Social Grade	**C2DE *(Ref=ABC1)***	0.76	0.54	1.06	0.90	0.60	1.36
Age group	**18-24 *(Ref=25-64)***	1.40	0.69	2.86	1.00	0.40	2.49
	**65+ *(Ref=25-64)***	0.98	0.67	1.43	0.78	0.49	1.26
Ethnicity	**BAME *(Ref=White)***	0.91	0.43	1.93	0.72	0.27	1.91
Dog Ownership	**Yes *(Ref=No)***	1.33	0.92	1.91	1.15	0.74	1.80

Supplementary Table 5: Multinomial logistic regression models of being in each outcome group for levels of agreement that respondents do more physical activity in green space following the movement restrictions, results are reported as Risk Ratios (RR) with 95% Lower Confidence Limits (LCL) and Upper Confidence Limits (UCL); **p<0.05**.

		RR of Agree to "Neither agree or disagree" and 95% CIs			RR of Disagree to "Neither agree or disagree" and 95% CIs		
		RR	LCL	UCL	RR	LCL	UCL
	**Intercept**	1.09	0.78	1.51	1.22	0.89	1.66
Sex	**Female *(Ref=Male)***	1.44	0.99	2.08	1.35	0.97	1.88
Social Grade	**C2DE *(Ref=ABC1)***	0.75	0.51	1.09	0.85	0.61	1.19
Age group	**18-24 *(Ref=25-64)***	2.07	0.99	4.36	1.13	0.52	2.49
	**65+ *(Ref=25-64)***	**0.51**	**0.32**	**0.80**	0.93	0.64	1.34
Ethnicity	**BAME *(Ref=White)***	0.95	0.44	2.08	**0.38**	**0.15**	**0.97**
Dog Ownership	**Yes *(Ref=No)***	**0.43**	**0.28**	**0.66**	0.95	0.68	1.35

Supplementary Table 6: Change in green space visit frequency since restrictions by individual demographic and socio-economic characteristics (unweighted).

	**Visited green space in the year before restrictions**		**Visited green space after restrictions (only those who visited green space before restrictions)**		**Green space visitation change since lockdown**		
	**Yes (N=2045, 93.5%)**	**No (N=142, 6.4%)**	**Yes (N=1099, 53.8%)**	**No (N=943, 46.2%)**	**Decreased (N=1392, 63.3%)**	**Same (N=473, 21.5%)**	**Increased (N=333, 15.2%)**
	% (N)	*% (N)*	*% (N)*	*% (N)*	*% (N)*	*% (N)*	*% (N)*
Sex							
**Male (ref)**	92.6% *(919)*	7.4% *(73)*	55.2% *(507)*	44.8% *(411)*	61.6% *(609)*	23.5% *(232)*	15% *(148)*
**Female**	94.2% *(1126)*	5.8% *(69)*	52.7% *(592)*	47.3% *(532)*	64.8% *(783)*	19.9% *(241)*	15.3% *(185)*
Social grade							
**ABC1 (ref)**	95.8% *(1328)*	4.2% *(58)*	56.7% *(753)*	43.3% *(574)*	62.9% *(871)*	18.8% *(260)*	18.3% *(254)*
**C2DE**	89.5% *(717)*	10.5% *(84)*	48.4% *(346)*	51.6% *(369)*	64.1% *(521)*	26.2% *(213)*	9.7% *(79)*
Age							
**18-24**	93.3% *(139)*	6.7% *(10)*	56.9% *(78)*	43.1% *(59)*	58.4% *(87)*	20.8% *(31)*	20.8% *(31)*
**25-64 (ref)**	93.6% *(1364)*	6.4% *(94)*	56.1% *(765)*	43.9% *(598)*	61.8% *(906)*	21.5% *(315)*	16.7% *(245)*
**65+**	93.4% *(542)*	6.6% *(38)*	47.2% *(256)*	52.8% *(286)*	68.4% *(399)*	21.8% *(127)*	9.8% *(57)*
Ethnicity							
**White (ref)**	93.6% *(1592)*	6.4% *(109)*	52.1% *(828)*	47.9% *(762)*	64.1% *(1098)*	22.5% *(385)*	13.4% *(229)*
**BAME**	84.1% *(69)*	15.9% *(13)*	47.8% *(33)*	52.2% *(36)*	57% *(45)*	30.4% *(24)*	12.7% *(10)*
Dog ownership							
**Don't own a dog/s (ref)**	93.1% *(1476)*	6.9% *(109)*	51.4% *(757)*	48.6% *(717)*	64.4% *(1027)*	19.5% *(311)*	16.1% *(256)*
**Own a dog/s**	94.5% *(569)*	5.5% *(33)*	60.2% *(342)*	39.8% *(226)*	60.4% *(365)*	26.8% *(162)*	12.7% *(77)*

Don't know/ can't recall responses were all excluded from analysis.

Supplementary Table 7: Change in green space experience since restrictions by individual demographic and socio-economic characteristics (unweighted).

	**Using green spaces benefits my mental health more now**			**Miss social interaction in green spaces now**			**Do more physical activity in green spaces now**		
	**Agree (N=721, 65.8%)**	**Neither (N=272, 24.8%)**	**Disagree (N=102, 9.3%)**	**Agree (N=590, 54.1%)**	**Neither (N=288, 26.4%)**	**Disagree (N=213, 19.5%)**	**Agree (N=322, 29.5%)**	**Neither (N=337, 30.9%)**	**Disagree(N=431, 39.5%)**
	% (N)		*% (N)*	*% (N)*		*% (N)*	*% (N)*		*% (N)*
Sex									
**Male (ref)**	59.6% *(300)*	30.8% *(155)*	9.5% *(48)*	45.8% *(231)*	30% *(151)*	24.2% *(122)*	27.4% *(137)*	35.4% *(177)*	37.2% *(186)*
**Female**	71.1% *(421)*	19.8% *(117)*	9.1% *(54)*	61.2% *(359)*	23.3% *(137)*	15.5% *(91)*	31.4% *(185)*	27.1% *(160)*	41.5% *(245)*
Social grade									
**ABC1 (ref)**	68% *(511)*	22.9% *(172)*	9.1% *(68)*	54.9% *(412)*	25.3% *(190)*	19.7% *(148)*	31.6% *(236)*	28.9% *(216)*	39.6% *(296)*
**C2DE**	61% *(210)*	29.1% *(100)*	9.9% *(34)*	52.2% *(178)*	28.7% *(98)*	19.1% *(65)*	25.1% *(86)*	35.4% *(121)*	39.5% *(135)*
Age									
**18-24**	67.5% *(52)*	19.5% *(15)*	13% *(10)*	64.9% *(50)*	22.1% *(17)*	13% *(10)*	48.7% *(38)*	16.7% *(13)*	34.6% *(27)*
**25-64 (ref)**	69% *(526)*	21% *(160)*	10% *(76)*	53.7% *(407)*	25.9% *(196)*	20.4% *(155)*	30.8% *(233)*	30.1% *(228)*	39.1% *(296)*
**65+**	55.9% *(143)*	37.9% *(97)*	6.2% *(16)*	52% *(133)*	29.3% *(75)*	18.8% *(48)*	20% *(51)*	37.6% *(96)*	42.4% *(108)*
Ethnicity									
**White (ref)**	62.8% *(518)*	27.3% *(225)*	9.9% *(82)*	51.6% *(423)*	28% *(230)*	20.4% *(167)*	26.5% *(218)*	32.3% *(266)*	41.2% *(339)*
**BAME**	75% *(24)*	21.9% *(7)*	3.1% *(1)*	51.5% *(17)*	30.3% *(10)*	18.2% *(6)*	38.7% *(12)*	38.7% *(12)*	22.6% *(7)*
Dog ownership									
**Don't own a dog/s (ref)**	68.2% *(514)*	23.9% *(180)*	8% *(60)*	52.5% *(395)*	27.1% *(204)*	20.5% *(154)*	34.5% *(259)*	28.7% *(215)*	36.8% *(276)*
**Own a dog/s**	60.7% *(207)*	27% *(92)*	12.3% *(42)*	57.7% *(195)*	24.9% *(84)*	17.5% *(59)*	18.5% *(63)*	35.9% *(122)*	45.6% *(155)*

Don't know/ can't recall responses were all excluded from analysis.
