# Supplementary material for: Change in time spent visiting and experiences of green space following restrictions on movement during the COVID-19 pandemic: a nationally representative cross-sectional study of UK adults
Source: BMJ Open. 2021 Mar 8;11(3):e044067. doi: 10.1136/bmjopen-2020-044067 (PMC7942249; doi:10.1136/bmjopen-2020-044067)
Supplement: Author's manuscript [file bmjopen-2020-044067.draft_revisions.pdf]

BMJ Open

BMJ Open is committed to open peer review. As part of this commitment we make the peer review history of every article we publish publicly available.

When an article is published we post the peer reviewers' comments and the authors' responses online. We also post the versions of the paper that were used during peer review. These are the versions that the peer review comments apply to.

The versions of the paper that follow are the versions that were submitted during the peer review process. They are not the versions of record or the final published versions. They should not be cited or distributed as the published version of this manuscript.

BMJ Open is an open access journal and the full, final, typeset and author-corrected version of record of the manuscript is available on our site with no access controls, subscription charges or pay-per-view fees (http://bmjopen.bmj.com).

If you have any questions on BMJ Open's open peer review process please email info.bmjopen@bmj.com

BMJ Open

Changes in the frequency and experiences of visits to green space following restrictions on movement during the COVID-19 pandemic: A nationally representative cross-sectional study of UK adults.

Journal:	*BMJ Open*
Manuscript ID	bmjopen-2020-044067
Article Type:	Original research
Date Submitted by the Author:	21-Aug-2020
Complete List of Authors:	Burnett, Hannah; University of Glasgow, MRC/CSO Social and Public Health Sciences Unit<br>Olsen, Jonathan; University of Glasgow, MRC/CSO Social and Public Health Sciences Unit<br>Nicholls, Natalie; University of Glasgow, MRC/CSO Social and Public Health Sciences Unit<br>Mitchell, Richard; University of Glasgow, MRC/CSO Social and Public Health Sciences Unit
Keywords:	MENTAL HEALTH, COVID-19, PUBLIC HEALTH

SCHOLARONE™
Manuscripts

BMJ

I, the Submitting Author has the right to grant and does grant on behalf of all authors of the Work (as defined in the below author licence), an exclusive licence and/or a non-exclusive licence for contributions from authors who are: i) UK Crown employees; ii) where BMJ has agreed a CC-BY licence shall apply, and/or iii) in accordance with the terms applicable for US Federal Government officers or employees acting as part of their official duties; on a worldwide, perpetual, irrevocable, royalty-free basis to BMJ Publishing Group Ltd ("BMJ") its licensees and where the relevant Journal is co-owned by BMJ to the co-owners of the Journal, to publish the Work in this journal and any other BMJ products and to exploit all rights, as set out in our licence.

The Submitting Author accepts and understands that any supply made under these terms is made by BMJ to the Submitting Author unless you are acting as an employee on behalf of your employer or a postgraduate student of an affiliated institution which is paying any applicable article publishing charge ("APC") for Open Access articles. Where the Submitting Author wishes to make the Work available on an Open Access basis (and intends to pay the relevant APC), the terms of reuse of such Open Access shall be governed by a Creative Commons licence – details of these licences and which Creative Commons licence will apply to this Work are set out in our licence referred to above.

Other than as permitted in any relevant BMJ Author's Self Archiving Policies, I confirm this Work has not been accepted for publication elsewhere, is not being considered for publication elsewhere and does not duplicate material already published. I confirm all authors consent to publication of this Work and authorise the granting of this licence.

For peer review only

Changes in the frequency and experiences of visits to green space following restrictions on movement during the COVID-19 pandemic: A nationally representative cross-sectional study of UK adults.

Hannah Burnett[1], Jonathan R Olsen[1], Natalie Nicholls[1], Richard Mitchell[1]
[1]MRC/CSO Social and Public Health Sciences Unit, University of Glasgow, Berkeley Square, 99 Berkeley Street, Glasgow, G3 7HR, United Kingdom.

Correspondence:
Hannah Burnett
MRC/CSO Social and Public Health Sciences Unit,
University of Glasgow,
Berkeley Square,
99 Berkeley Street,
Glasgow,
G3 7HR.
Email: h.burnett.1@research.gla.ac.uk

Key words: mental health; COVID-19; public health.

Word Count: 3,054
Tables : 3
Figures : 1
Supplementary tables : 3

ABSTRACT

Objectives Green space has previously been found to positively influence health and well-being, however inequalities in green space visitation are prevalent. Restrictions on movement enforced due to the COVID-19 pandemic could have exacerbated the existing inequalities regarding who visits green space. Therefore, this study aimed to explore how restrictions on movement have changed the frequency and experiences of visits to green space in the United Kingdom (UK) and how these differed by individual-level demographic and socio-economic characteristics.

Design and outcome measures A nationally representative cross-sectional survey administered through YouGov between 30th April – 1st May 2020. Data were collected on the frequency of visits and experiences within green space, including missing social interaction, increased physical activity, and feeling greater mental health benefits in green space. Demographic information was collected on sex, age, ethnicity, social grade and dog ownership. Associations between specific outcome variables and predictors were assessed using logistic regression.

Setting United Kingdom, with population weights applied.

Participants 2,252 adults aged 18 years and over.

Results Overall, 63% of respondents reported a decrease in green space visits following restrictions on movement. Lower social grade respondents were less likely to visit green space before *and* after restrictions were enforced (OR: 0.35 (95% CI: 0.24-0.51); OR: 0.68 (95% CI: 0.56-0.83)). Older (65+) (OR: 1.30 (95% CI: 1.04-1.64)) and female (OR: 1.26 (95% CI:1.04-1.54)) respondents were more likely than middle age (25 to 64 years) and male respondents to report decreased visits and to agree that green space benefitted their mental health more following restrictions on movement.

Conclusions Inequalities in green space use were sustained, and possibly exacerbated, during restrictions on movement. Further investigation is required to determine how these patterns change through the different stages of the COVID-19 pandemic in the UK.

(Word Count: 289)

Strengths and Limitations of this study

- Our data are currently the only existing data covering change in the frequency and experiences of green space for the UK population following the restrictions on movement enforced due to the COVID-19 pandemic.
- The sample is nationally representative of UK adults.
- The data collected for this study were from a cross-sectional survey, therefore it is not possible to demonstrate causality between change in green space use and experiences and the demographic data.
- Certain variable response categories were recategorised for analysis, which meant that some distinctiveness across groups were lost, particularly for the ethnicity and age variables.

INTRODUCTION

Evidence suggests that exposure to green space has a positive influence on health and well-being.[1,2] Green space has been found to increase levels of social interaction and physical activity, and decrease levels of all-cause mortality, loneliness and stress.[3–5] Additionally, there is evidence that contact with green space may disproportionately benefit disadvantaged populations, reducing health inequalities and therefore weakening the effects of poverty – known as the 'equigenic' effect.[6,7]

The health impacts of contact with green spaces are quite well studied by both observational and experimental designs. There is rather less literature on the levels, motivations and barriers of visiting green spaces in the first place. Recent surveys by Natural England and Scottish Natural Heritage have found that 74% of the English population and 70% of the Scottish population visit green space 'frequently' (once a month or more often). In both countries, the top three reasons stated for frequent green space use were health and exercise, walking the dog, and to relax/unwind.[8,9] Further research exploring the motivations and reasons for visiting green space found that visitation frequency is affected by an individual's feelings of nature connectedness, as well as their childhood exposure to nature.[10–12]. It is important to note that substantial inequalities in green space use have also been reported, with existing studies stating that females, older people and those from less advantaged socioeconomic positions (SEP) are more likely to be infrequent users than their male, younger and higher SEP counterparts.[9,13–15]

In 2020, the United Kingdom (UK) experienced major disruption to everyday life due to the novel coronavirus disease 2019 (COVID-19) pandemic. COVID-19 is an infectious disease first identified in the city of Wuhan, China in December 2019.[16] As a result of the COVID-19 outbreak, the UK announced a series of restrictions on movement from 23rd March 2020. These included rules around social distancing, meaning that people could only leave their households to make 'essential trips' for food, medication and exercise.[17] The aim of this research was to explore changes in the frequency and experience of visits to green space among the UK population following restrictions on movement. The sociodemographic characteristics focused on were those identified by the literature as being most consistent markers of inequality in use. The research questions were:

1) How did frequency of green space use change following restrictions on movement, compared with before, and for whom?
2) How did experiences of visits to green space change following the restrictions, and did any change vary by sex, social grade, age, ethnicity and dog ownership? Specifically,
   a) Did respondents feel that green space benefitted their mental health more since restrictions than before?
   b) Did respondents miss social interaction in green space more following restrictions on movement?
   c) Had physical activity increased following restrictions on movement?

METHODS

Survey design and sample

An online cross-sectional survey was administered by YouGov between 30th April and 1st May 2020.[18] Questions were answered by a sample of 2,252 adults from the UK (aged 18 and over). The sample was drawn from a panel of over 800,000 individuals who specifically opted in to participate in online research activities. Sample members were selected at random from the panel and sent an email providing a survey link. Table 1 shows the themes and specific survey instruments analysed in this paper. Demographic information about participants was also collected (including sex, age and social grade, which was classified by occupation).[19,20] Although the sample was reasonably large, small numbers in variable response categories necessitated some category mergers (Table 1 and Supplementary Table 1).

The survey covered adults from across the UK, with respondents from England (N=1,875), Scotland (N=209), Wales (N=107), Northern Ireland (N=61) and weightings were applied to render the sample representative of UK adults (detailed below). When the survey was distributed, the same restrictions on movement were implemented across the UK. Since then, the individual parliaments/assemblies representing these countries have imposed different COVID-19 policies.

Table 1: Survey themes and specific items analysed, including variables recategorised.

Themes	Question/statement	Potential responses	Recategorised
Visit frequency and change	*"Did you EVER visit a green space in the year before the movement restrictions were enforced in the UK?"*	Yes, I did<br>No, I didn't<br>Don't know/ can't recall	-
	"Please think about your behaviour since the UK enforced a 'lock down' to restrict movement, as a result of the current Coronavirus (COVID-19) outbreak (i.e. since 23rd March 2020). Have you visited a green space since the movement restrictions have been enforced in the UK?"	Yes, I have<br>No, I haven't<br>Don't know/ can't recall	-
	"How much, if at all, has the amount of time that you have spent visiting green spaces changed since the 'lock down' movement restrictions began (i.e. 23rd March 2020) compared to before?"	Increased a lot<br>Increased a little<br>No difference<br>Decreased a little<br>Decreased a lot<br>Don't know	'Increased' (increased a lot and increased a little vs. no difference, decreased a little and decreased a lot)<br><br>'Decreased' (decreased a lot and decreased a little vs. no difference, increased a little and increased a lot)
Experience change (If respondent had visited a green space since the movement	*"I feel that being in green spaces benefits my mental health <u>more now</u>, than before the movement restrictions were in place"*	Strongly agree<br>Slightly agree<br>Neither agree nor disagree<br>Slightly disagree<br>Strongly disagree<br>Don't know/ can't recall	'Agree' (strongly agree and slightly agree vs. neither agree nor disagree, slightly disagree, and strongly disagree)<br><br>'Disagree' (slightly disagree and strongly disagree vs. neither agree nor disagree, slightly agree, and strongly agree)

restrictions were enforced)	*"I have missed seeing and/ or talking with people in green spaces since the movement restrictions were introduced"*	Strongly agree<br>Slightly agree<br>Neither agree nor disagree<br>Slightly disagree<br>Strongly disagree<br>Don't know/ can't recall	'Agree' (strongly agree and slightly agree vs. neither agree nor disagree, slightly disagree, and strongly disagree)<br><br>'Disagree' (slightly disagree and strongly disagree vs. neither agree nor disagree, slightly agree, and strongly agree)
	"I do more physical activity in green spaces now, than I did before the movement restrictions were introduced"	Strongly agree<br>Slightly agree<br>Neither agree nor disagree<br>Slightly disagree<br>Strongly disagree<br>Don't know/ can't recall	'Agree' (strongly agree and slightly agree vs. neither agree nor disagree, slightly disagree, and strongly disagree)<br><br>'Disagree' (slightly disagree and strongly disagree vs. neither agree nor disagree, slightly agree, and strongly agree)

Demographic variables

Individual level demographic and socio-economic characteristics were captured from the survey, as follows: sex (male, female); age group (18-24 years, 25-64 years, 65+ years); ethnicity (White, Black, Asian and minority ethnic (BAME)); dog ownership (Yes, No); and social grade (ABC1, C2DE), derived by YouGov from combined occupational social grade categories. ABC1 was the higher social grade, and included non-manual workers, for example, senior managers and owners of small establishments. C2DE was the lower social grade, and included all manual workers, for example, shop assistants and labourers.[21] Hereafter, social grade will be described as higher and lower social grade.

Patient and Public Involvement

There was no direct patient or public involvement in this study.

Analyses

Descriptive statistics

The count and proportion of respondents who had: visited green space before and after restrictions on movement were enforced; increased or decreased visitation; and agreed or disagreed with the three change in experience statements (Table 1) are presented. These were also explored by sex, age, ethnicity, dog ownership and social grade.

Statistical analysis

Multiple binary logistic regression analyses were conducted to assess the association between the individual predictor variables and each of the following outcomes:

1. having visited green space in the year before restrictions on movement were enforced;
2. having visited green space since restrictions on movement were enforced;

3. having increased green space visitation following restrictions on movement;
4. having decreased green space visitation following restrictions on movement;
5. agreeing that green space benefits their mental health more now (since restrictions on movement were enforced compared to before);
6. agreeing that they miss seeing/talking to people in green space now (since restrictions on movement were enforced compared to before); and
7. agreeing that they do more physical activity in green space now (since restrictions on movement were enforced compared to before).

Associations were expressed as odds ratios (OR) with 95% confidence intervals (CIs). Univariate models for each predictor were conducted first, followed by a fully adjusted model containing all predictors for each outcome. Weightings were calculated by YouGov and applied during analyses to render the sample representative of UK adults.[20] All analyses were conducted in R v3.5.1,[22] a full R script is available on GitHub.[23]

Interactions

Given existing literature,[9,13,24,25] we expected some interactions between predictor variables, which included sex, age, social grade, ethnicity and dog ownership, in their relationships with change in frequency and experiences within green spaces following restrictions on movement. We therefore explored interactions, adding interaction terms to the models, assessing their significance via Wald tests, and then producing predicted probabilities to aid interpretation of the significant interactions.

RESULTS

Descriptive statistics

93% of all respondents had visited green space in the year before restrictions on movement were enforced (Supplementary Table 2). In contrast, 49% of respondents reported visiting green space following restrictions on movement. 63% of respondents reported that the amount of time they spent in green space had decreased since restrictions on movement compared to before, with 15% reporting an increase, and 22% reporting no difference in visitation frequency.

A greater proportion of respondents agreed (65%) than disagreed (35%) that green space benefited their mental health more following restrictions on movement compared to before (Supplementary Table 3). More respondents agreed (54%) that they missed social interaction in green space more since restrictions on movement, than disagreed (46%). 71% of respondents disagreed that they had increased physical activity in green space since restrictions on movement, compared to 29% who agreed.

Visit frequency and change

Visiting green space before restrictions on movement

In the adjusted logistic regression model for visiting green space in the year before restrictions on movement were enforced (Table 2), only two variables had significant associations ($p<0.05$). Lower social grade respondents (OR: 0.35 (95%CI: 0.24-0.51)) and

BAME (OR: 0.43 (95%CI: 0.23-0.80)) respondents both had lower odds of visiting green space before restrictions on movement were enforced, compared to higher social grade and white respondents.

Visiting green space following restrictions on movement

Respondents in the lower social grade group were less likely to have visited green space than respondents in the higher social grade group (OR: 0.68 (95%CI: 0.56-0.83)) (Table 2). Respondents who owned a dog/s were more likely than respondents who did not to have visited green space after restrictions on movement were enforced (OR: 1.44 (95%CI: 1.16-1.79)).

Change in frequency of green space visitation

Lower social grade respondents were less likely than higher social grade respondents to report increased visits following restrictions on movement (OR: 0.52 (95%CI: 0.38-0.70)) (Table 2). Older respondents (aged 65+) were less likely to report increased green space visitations than respondents aged between 25-64 years (OR: 0.59 (95%CI: 0.41-0.84)). Sex and older age were significantly associated with *decreased* visits. Females were more likely to report decreased green space visits compared to males (OR: 1.26 (95%CI:1.04-1.54)), and older respondents were more likely to report a decrease in green space visits than respondents aged 25-64 years (OR: 1.30 (95%CI: 1.04-1.64)) (Table 2).

Interaction effects

Three interactions were significant ($p<0.05$); one between social grade and ethnicity in their association with visiting green space before restrictions on movement, and two between age and ethnicity, in their association with visiting green spaces since restrictions on movement and with *increased* visits since restrictions on movement.

Higher social grade white respondents had the highest probability of visiting green space before lockdown, but lower social grade BAME respondents were more likely to have visited green space before lockdown than the higher social grade BAME respondents (Figure 1). Young BAME respondents had a higher probability of visiting green space and reporting increased visits since lockdown compared to white and older BAME respondents. However, the number of BAME respondents, especially young and older BAME respondents, in this sample was small and these results should be interpreted with caution.

Table 2: Adjusted binary logistic regression models predicting change in frequency of green space visitation. OR = odds ratio; CI = confidence intervals; *p<0.05.*

	**Visited green space in the year before restrictions ('yes')**		**Visited green space after restrictions ('yes')**		**Green space visitation change since lockdown ('increased')**		**Green space visitation change since lockdown ('decreased')**	
	Adjusted		Adjusted		Adjusted		Adjusted	
	OR (95% CI)	p-value	OR (95% CI)	p-value	OR (95% CI)	p-value	OR (95% CI)	p-value
Sex								
**Male (ref)**	-	-	-	-	-	-	-	-
**Female**	1.35 (0.94-1.95)	0.109	0.92 (0.76-1.11)	0.361	0.96 (0.72-1.27)	0.755	1.26 (1.04-1.54)	*0.020*
Social grade								
**ABC1 (ref)**	-	-	-	-	-	-	-	-
**C2DE**	0.35 (0.24-0.51)	*<0.001*	0.68 (0.56-0.83)	*<0.001*	0.52 (0.38-0.70)	*<0.001*	0.96 (0.79-1.17)	0.704
Age								
**18-24**	2.92 (1.00-8.57)	0.051	0.93 (0.63-1.36)	0.692	1.60 (0.97-2.63)	0.064	0.77 (0.52-1.15)	0.201
**25-64 (ref)**	-	-	-	-	-	-	-	-
**65+**	1.22 (0.80-1.85)	0.353	0.81 (0.65-1.01)	0.058	0.59 (0.41-0.84)	*0.004*	1.30 (1.04-1.64)	*0.023*
Ethnicity								
**White (ref)**	-	-	-	-	-	-	-	-
**BAME**	0.43 (0.23-0.80)	*0.007*	0.91 (0.59-1.39)	0.651	1.15 (0.63-2.08)	0.650	0.67 (0.43-1.03)	0.068
Dog ownership								
**Don't own a dog/s (ref)**	-	-	-	-	-	-	-	-
**Own a dog/s**	1.29 (0.83-2.00)	0.260	1.44 (1.16-1.79)	*0.001*	0.74 (0.53-1.03)	0.070	0.83 (0.67-1.04)	0.109

Change in visit experience

Mental health benefits

Agreeing that green space benefits mental health more since restrictions on movement compared to before was associated with sex, social grade, age (only 65+) and dog ownership (Table 3). Females were more likely to agree than males that being in green space benefitted their mental health more following restrictions on movement than before (OR: 1.60 (95% CI: 1.19-2.15)). Whilst lower social grade (OR: 0.69 (95% CI: 0.51-0.93)), older respondents (OR: 0.58 (95% CI: 0.42-0.82)), and dog owners (OR: 0.72 (95% CI: 0.52-0.98)) were less likely to agree.

Missed social interaction

Female respondents were more likely to agree that they missed seeing and talking with other people in green space since restrictions on movement compared to before than male respondents (OR: 1.72 (95% CI: 1.30-2.28)) (Table 3).

Increased physical activity

Younger respondents were more likely (OR: 1.93 (95% CI: 1.06-3.54)) than the middle age group respondents to agree that they had done more physical activity since restrictions on movement were enforced whilst older respondents were less likely to agree (OR: 0.53 (95% CI: 0.35-0.79)) (Table 3). Respondents who owned a dog/s were less likely than respondents without a dog/s to agree (OR: 0.45 (95% CI: 0.31-0.65)).

DISCUSSION

There was a general polarisation of use following restrictions on movement; for example, older respondents reported decreased visits to green space, whilst younger respondents reported increased physical activity levels in green spaces following restrictions on movement. Our findings suggest that inequalities in use of green space between demographic groups were sustained following restrictions on movement, with lower social grade individuals less likely than higher social grade individuals to have visited green space before and since restrictions on movement. These inequalities were possibly exacerbated in the month after restrictions on movement were enforced, as females and older respondents were more likely to report a decrease in visits following restrictions on movement.

The proportion of respondents who visited green space before and following restrictions on movement decreased, from 93% to 49%. This was consistent with Natural England's findings from April 2020 where 49% of English adults reported green space visits in the previous two weeks.[26] Natural England conducted an online panel survey in April 2020 (N=2,083), the survey covered only the English population, collecting information on the frequency of time in green and natural spaces in the 12 months, and then 2 weeks, prior to the survey.[27] The survey did not directly ask respondents about whether they felt their experiences within green space had changed following restrictions on movement.

Table 3: Adjusted binary logistic regression models predicting change in experience of green space since restrictions. OR = odds ratio; CI = confidence intervals; *p<0.05.*

	**Using green spaces benefits my mental health more now ('agree')**		**Miss social interaction in green spaces now ('agree')**		**Do more physical activity in green spaces now ('agree')**	
	Adjusted		Adjusted		Adjusted	
	OR (95% CI)	p-value	OR (95% CI)	p-value	OR (95% CI)	p-value
Sex						
**Male (ref)**	-	-	-	-	-	-
**Female**	1.60 (1.19-2.15)	*0.002*	1.72 (1.30-2.28)	*<0.001*	1.22 (0.88-1.68)	0.232
Social grade						
**ABC1 (ref)**	-	-	-	-	-	-
**C2DE**	0.69 (0.51-0.93)	*0.014*	0.79 (0.59-1.06)	0.118	0.81 (0.58-1.14)	0.227
Age						
**18-24**	0.59 (0.32-1.11)	0.102	1.40 (0.77-2.57)	0.270	1.93 (1.06-3.54)	0.033
**25-64 (ref)**	-	-	-	-	-	-
**65+**	0.58 (0.42-0.82)	*0.002*	1.08 (0.78-1.50)	0.643	0.53 (0.35-0.79)	0.002
Ethnicity						
**White (ref)**	-	-	-	-	-	-
**BAME**	1.72 (0.80-3.69)	0.162	1.03 (0.53-2.01)	0.923	1.47 (0.73-2.96)	0.278
Dog ownership						
**Don't own a dog/s (ref)**	-	-	-	-	-	-
**Own a dog/s**	0.72 (0.52-0.98)	*0.038*	1.25 (0.92-1.70)	0.154	0.45 (0.30-0.65)	<0.001

For peer review only - http://bmjopen.bmj.com/site/about/guidelines.xhtml

Females and older individuals were more likely to have reported decreased visits to green space following restrictions on movement. These findings corroborate with existing data collected before restrictions on movement.[9,13,14] Boyd *et al.* analysed the Natural England ‘Monitor of Engagement with the Natural Environment’ survey (N=63,890) with a focus on infrequent use. They found that females and older adults in England were more likely to be infrequent visitors.[9] Cohen *et al.* explored physical activity levels in Los Angeles’ parks (N=1,318). They reported that age and sex were predictors of park use, with <5% of park users over 60 years old, and males using parks more often than females (62% vs. 38%).[13]

Our findings suggested that existing patterns of inequality in use by females were sustained and potentially exacerbated due to fears arising from the COVID-19 pandemic. For example, an Ipsos MORI survey was conducted in April 2020, collecting data on gender differences in British attitudes towards COVID-19. They found that women were more likely to report following government rules to avoid leaving their home (78%) than men (68%) and felt more uncomfortable about returning to “normal” (N=1,000).[28,29]

Previous studies have found that females feel more vulnerable than males in green spaces, especially without company.[14,15,24,30–32] There are few quantitative studies focusing on sex and the importance of social interaction as a motivation behind green space use. We found that females were more likely to agree that they missed seeing/talking with others in green space than males. This could be explained by the nature of the restrictions on movement at this time, particularly the inability to socially interact with individuals outside your household in green space.

Given older age is probably the most important risk factor for an adverse outcome from COVID-19,[33,34] it was not surprising that older individuals were more likely to report a decrease in green space visits and to disagree that they were doing more physical activity following restrictions on movement. These findings corroborate with the majority of research exploring older age and green space use before the restrictions on movement.[9,13,35] The significant decrease in green space visits following restrictions on movement for older respondents could be explained by the government advice for over 70s and those with an underlying health condition to shield, minimise interaction and stay at home, for around 12 weeks from 21st March 2020.[36]

Lower social grade respondents reported little or no change in visitation to green space, with visit frequency remaining low both before and after restrictions on movement. This is supported by previous studies exploring the association between socio-economic position (SEP) and green space use.[9,15,35] One possible explanation is a lack of interest in visiting green space reported by lower SEP individuals.[9] Additionally, the social grade variable was categorised by occupation, and it was likely that individuals categorised as lower social grade were working in manual or service occupations.[21] They may have continued at their usual workplace/working hours during the COVID-19 pandemic. Findings from the Office for National Statistics support this explanation, with 30.5% of employees in the bottom three income deciles (monthly earnings of up to £1,450) considered as key workers in March/April 2020, compared to 26.4% in the top three income deciles (monthly earnings of up to £3,250).[37]

Younger BAME respondents had a higher probability of visiting green space following restrictions on movement and reporting increased visits since restrictions on movement, compared to white and older BAME respondents. A survey on park use and ethnic integration

in Reading, UK (N=294) reported similar findings, with Black and Asian youths accounting for a higher proportion of parks users than white youths.[38] Further exploration is required to enable interpretation of these interaction results because of our small BAME sample.

Strengths and limitations

Our study has several strengths; to our knowledge, currently this is the only data covering UK population change in use and experiences within green space at the start of the restrictions on movement from 23rd March 2020. This means that the data could provide timely information to local and national governments across the UK. The rapid collection of data, just over a month after the restrictions on movement were introduced, reduces risk of recall bias. The sample is nationally representative of UK adults, with weightings calculated by YouGov and applied to all analyses, reducing risk of selection bias.

A further strength of our study is providing quantitative data on the importance of social interaction as a driver for using green space, which is under-reported in the literature. We analysed social grade at an individual level, which provides greater detail and accuracy of the respondent's SEP than at neighbourhood level.

There are some caveats. The survey design was cross-sectional, and we are unable to demonstrate causality between change in green space use and experiences and the demographic data. Additionally, the data on use and experiences are self-reported and therefore subjective. Certain variable response categories had to be recategorised in order to analyse them. This is particularly evident in the ethnicity and age variables, where some distinctiveness across groups was lost.

We encourage future research to further investigate the interactions between ethnicity, age and social grade in relation to green space use to enable more substantial interpretation of the results and to explore whether the patterns are consistent over time and in different contexts. Moreover, further research is required to explore the reasons *why* many patterns and changes in use of green space since restrictions on movement have emerged.

CONCLUSION

During the COVID-19 pandemic, our study provides timely and novel evidence to suggest that green space use and experiences were affected during the first month of restrictions on movement in the UK. Our findings suggested that inequalities in use were sustained, with lower social grade individuals less likely to visit green space than higher social grade individuals before and following restrictions on movement were enforced. It is possible that these inequalities were exacerbated, as females and older individuals were the groups most likely to report decreased visits following restrictions on movement. Further investigation is required to support these findings and how they may change through the stages of the COVID-19 pandemic and the relaxing, and potential re-introduction, of restrictions on movement.

COMPETING INTERESTS
None declared.

DATA SHARING STATEMENT

For peer review only - http://bmjopen.bmj.com/site/about/guidelines.xhtml

We have made our research dataset publicly available (Datacite DOI: 10.5525/gla.researchdata.1038 - embargoed until 31/10/2023).

CONTRIBUTORS

HB, JRO and RM designed the study and survey; HB conducted data analysis which was reviewed by NN; HB wrote the first draft of the paper; all authors contributed to interpreting the results and revising the draft. The corresponding author attests that all listed authors meet authorship criteria and that no others meeting the criteria have been omitted.

FUNDING STATEMENT

The authors (HB, JRO, NN & RM) are part of the Places and Health Programme at the MRC/CSO Social and Public Health Sciences Unit (SPHSU), University of Glasgow, supported by the Medical Research Council (MC_UU_12017/10) and the Chief Scientist Office (SPHSU10).

References:

1 Barton J, Pretty J. What is the Best Dose of Nature and Green Exercise for Improving Mental Health? A Multi-Study Analysis. *Environ Sci Technol* 2010;**44**:3947–55. doi:10.1021/es903183r

2 Douglas O, Lennon M, Scott M. Green space benefits for health and well-being: A life-course approach for urban planning, design and management. *CITIES* 2017;**66**:53–62. doi:10.1016/j.cities.2017.03.011

3 Twohig-Bennett C, Jones A. The health benefits of the great outdoors: A systematic review and meta-analysis of greenspace exposure and health outcomes. *Environ Res* 2018;**166**:628–37. doi:10.1016/j.envres.2018.06.030

4 Maas J, Van Dillen SME, Verheij RA, *et al.* Social contacts as a possible mechanism behind the relation between green space and health. *Health Place* 2009;**15**:586–95. doi:10.1016/j.healthplace.2008.09.006

5 Wells N, Evans G. NEARBY NATURE A Buffer of Life Stress Among Rural Children. *Environ Behav* 2003;**35**:311–30. doi:10.1177/0013916503251445

6 Frumkin H, Bratman GN, Breslow SJ, *et al.* Nature Contact and Human Health: A Research Agenda. *Environ Health Perspect* 2017;**125**. doi:10.1289/EHP1663

7 Mitchell RJ, Richardson EA, Shortt NK, *et al.* Neighborhood Environments and Socioeconomic Inequalities in Mental Well-Being. *Am J Prev Med* 2015;**49**:80–4. doi:10.1016/j.amepre.2015.01.017

8 Wilson V, Seddon B. Scotland's People and Nature Survey 2017/18 – outdoor recreation and health modules. (Scottish Natural Heritage Research Report No. 1062). Inverness: 2018.

9 Boyd F, White MP, Bell SL, *et al.* Who doesn't visit natural environments for recreation and why: A population representative analysis of spatial, individual and temporal factors among adults in England. *Landsc Urban Plan* 2018;**175**:102–13. doi:10.1016/j.landurbplan.2018.03.016

10 Bell SL, Phoenix C, Lovell R, *et al.* Green space, health and wellbeing: making space for individual agency. *Health Place* 2014;**30**:287–92. doi:10.1016/j.healthplace.2014.10.005

11 Lin BB, Fuller RA, Bush R, *et al.* Opportunity or Orientation ? Who Uses Urban Parks and Why. 2014;**9**:1–7. doi:10.1371/journal.pone.0087422

12 Ward Thompson C, Aspinall P, Montarzino A. The Childhood Factor Adult Visits to Green Places and the Significance of Childhood Experience. *Environ Behav*

2008;**40**:111–43. doi:10.1177/0013916507300119
13 Cohen DA, McKenzie TL, Sehgal A, *et al.* Contribution of Public Parks to Physical Activity. *Am J Public Health* 2007;**97**:509–14. doi:10.2105/AJPH.2005.072447
14 Lee ACK, Maheswaran R. The health benefits of urban green spaces: A review of the evidence. *J Public Health (Bangkok)* 2011;**33**:212–22. doi:10.1093/pubmed/fdq068
15 Morris J, O'Brien E, Ambrose-Oji B, *et al.* Access for all? Barriers to accessing woodlands and forests in Britain. *Local Environ Int J Justice Sustain* 2011;**16**:375–96. doi:10.1080/13549839.2011.576662
16 Wynants L, Van Calster B, Bonten MMJ, *et al.* Prediction models for diagnosis and prognosis of covid-19 infection: systematic review and critical appraisal . *BMJ* 2020;**369**. doi:10.1136/bmj.m1328
17 Johnson B. Prime Minister's statement on coronavirus (COVID-19): 23 March 2020. GOV.UK. 2020.https://www.gov.uk/government/speeches/pm-address-to-the-nation-on-coronavirus-23-march-2020 (accessed 7 Jul 2020).
18 YouGov. About YouGov Company. https://yougov.co.uk/about/ (accessed 8 Jun 2020).
19 YouGov and ESOMAR (World Association for Social Opinion and Market Research). ESOMAR 28: 28 Questions to help Online Research Buyers. The Netherlands: 2012. http://cdn.yougov.com/cumulus_uploads/document/t3r5k565j5/ESOMAR_28.pdf (accessed 8 Jun 2020).
20 YouGov. Panel Methodology. https://yougov.co.uk/about/panel-methodology/ (accessed 8 Jun 2020).
21 The Market Research Society (MRS). Definitions employed in Social Grading. MRS. https://www.mrs.org.uk/pdf/Definitions used in Social Grading based on OG7.pdf (accessed 2 Jul 2020).
22 R Core Team. R: A Language and Environment for Statistical Computing. 2018.https://www.r-project.org/
23 Burnett H. Change in green space use and experiences following UK restrictions on movement. GitHub. 2020.https://github.com/hannahburnett/greenspace-covid_code (accessed 21 Jul 2020).
24 Richardson EA, Mitchell R. Gender differences in relationships between urban green space and health in the United Kingdom. *Soc Sci Med* 2010;**71**:568–75. doi:10.1016/J.SOCSCIMED.2010.04.015
25 Astell-Burt T, Mitchell R, Hartig T, *et al.* The association between green space and mental health varies across the lifecourse. A longitudinal study. *J Epidemiol Community Heal* 2014;**68**:578–83. doi:10.1136/jech-2013-203767
26 Natural England. The People and Nature Survey for England: Monthly interim indicators for April 2020 (Experimental Statistics) . Nat. Engl. 2020.https://www.gov.uk/government/publications/the-people-and-nature-survey-for-england-monthly-interim-indicators-for-april-2020-experimental-statistics/the-people-and-nature-survey-for-england-monthly-interim-indicators-for-april-2020-experimental-statistics (accessed 25 Jun 2020).
27 Natural England. The People and Nature Survey For England. 2020. http://publications.naturalengland.org.uk/publication/6382837173583872
28 Ipsos MORI. Gender differences in attitudes towards Coronavirus. 2020. https://www.ipsos.com/sites/default/files/ct/news/documents/2020-05/fawcett_society_presentation.pdf (accessed 10 Jul 2020).
29 Ipsos MORI, The Fawcett Society. 6 in 10 women finding it harder to stay positive day-to-day due to Coronavirus: Women more likely to bearing the emotional and impact of the pandemic . Fawcett Soc.

2020.https://www.fawcettsociety.org.uk/Handlers/Download.ashx?IDMF=f173fde3-4edb-4c51-8453-7244e867ed90 (accessed 10 Jul 2020).

30 Thompson CW, Aspinall P, Bell S, *et al.* "It Gets You Away From Everyday Life": Local Woodlands and Community Use—What Makes a Difference? *Landsc Res* 2007;**30**:109–46. doi:10.1080/0142639042000324794

31 Burgess J. Focusing on Fear: The Use of Focus Groups in a Project for the Community Forest Unit, Countryside Commission. 1996.

32 O'Brien L, Morris J. Well-being for all? The social distribution of benefits gained from woodlands and forests in Britain. *Local Environ Int J Justice Sustain* 2014;**19**:356–83. doi:10.1080/13549839.2013.790354

33 Public Health England. Disparities in the risk and outcomes from COVID-19. London: 2020. www.facebook.com/PublicHealthEngland (accessed 26 Jul 2020).

34 Office for National Statistics. Deaths registered weekly in England and Wales, provisional: week ending 10 July 2020. ONS. 2020.https://www.ons.gov.uk/peoplepopulationandcommunity/birthsdeathsandmarriages/deaths/bulletins/deathsregisteredweeklyinenglandandwalesprovisional/latest#deaths-registered-by-age-group (accessed 26 Jul 2020).

35 TNS. Scotland's People and Nature Survey 2013/14. (Scottish Natural Heritage Commissioned Report No. 679). 2014.

36 Johnson B. Prime Minister's statement on coronavirus (COVID-19): 16 March 2020. GOV.UK. 2020.https://www.gov.uk/government/speeches/pm-statement-on-coronavirus-16-march-2020 (accessed 26 Jun 2020).

37 Office for National Statistics. Coronavirus and how people spent their time under lockdown. ONS. 2020.https://www.ons.gov.uk/economy/nationalaccounts/satelliteaccounts/bulletins/coronavirusandhowpeoplespenttheirtimeunderrestrictions/28marchto26april2020 (accessed 16 Jul 2020).

38 Ravenscroft N, Markwell S. Ethnicity and the integration and exclusion of young people through urban park and recreation provision. *Manag Leis* 2000;**5**:135–50. doi:10.1080/13606710050084838

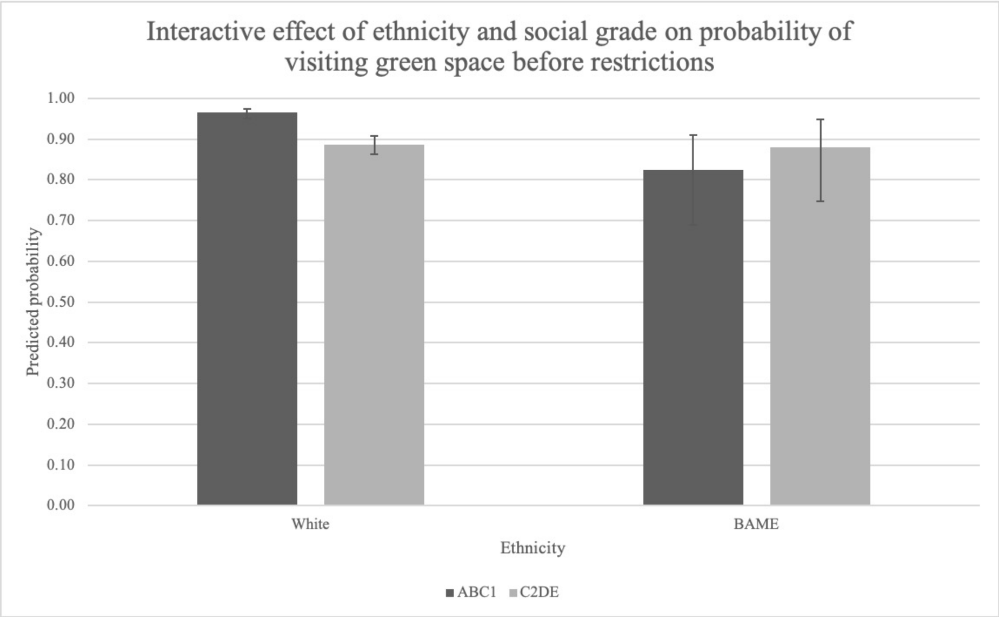

Figure 1: The significant interaction between social grade and ethnicity in their association with visiting green space before restrictions on movement, with 95% confidence interval bars.

202x124mm (144 x 144 DPI)

Supplementary Materials

Supplementary Table 1: Category mergers of the response categories (ethnicity, age, social grade, sex and dog ownership).

	Original categories (Unweighted N)	New categories (Unweighted N)
**Ethnicity**	White British (1659)	White (1747)
	Any other white background (86)	
	White and Black Caribbean (6)	BAME (87)
	White and Black African (4)	
	White and Asian (8)	
	Any other mixed background (8)	
	Indian (17)	
	Pakistani (5)	
	Bangladeshi (4)	
	Any other Asian background (3)	
	Black Caribbean (4)	
	Black African (3)	
	Any other black background (3)	
	Chinese (10)	
	Other ethnic group (12)	
	Prefer not to say (19)	Missing (418)
	Not answered (399)	
**Age**	18-24 (165)	18-24 (165)
	25-34 (316)	25-64 (1497)
	35-44 (396)	
	45-54 (398)	
	55-64 (387)	
	65+ (590)	65+ (590)
**Social grade**	ABC1 (1419)	
	C2DE (833)	
**Sex**	Male (1018)	
	Female (1234)	
**Dog ownership**	Yes (616)	
	No (1636)	

For peer review only

Supplementary Table 2: Change in green space visit frequency since restrictions by individual demographic and socio-economic characteristics (weighted).

	Visited green space in the year before restrictions		Visited green space after restrictions		Green space visitation change since lockdown		
	**Yes (N=2014; 93%)**	**No (N=158; 7%)**	**Yes (N=1086; 49%)**	**No (N=1123; 51%)**	**Increased (N=324; 15%)**	**Decreased (N=1375; 63%)**	**No difference (N=480; 22%)**
	% *(N)*	% *(N)*	% *(N)*	% *(N)*	% *(N)*	% *(N)*	% *(N)*
Sex							
**Male**	92% *(963)*	8% *(88)*	50% *(529)*	50% *(538)*	15% *(156)*	61% *(636)*	24% *(252)*
**Female**	94% *(1050)*	6% *(70)*	49% *(557)*	51% *(585)*	15% *(167)*	65% *(739)*	20% *(228)*
Social grade							
**ABC1**	95% *(1186)*	5% *(59)*	53% *(667)*	47% *(590)*	18% *(228)*	63% *(775)*	19% *(236)*
**C2DE**	89% *(828)*	11% *(99)*	44% *(419)*	56% *(533)*	10% *(95)*	64% *(600)*	26% *(244)*
Age							
**18-24**	91% *(203)*	9% *(21)*	50% *(115)*	50% *(117)*	19% *(43)*	57% *(126)*	24% *(54)*
**25-64**	93% *(1335)*	7% *(103)*	51% *(746)*	49% *(712)*	16% *(232)*	62% *(895)*	22% *(314)*
**65+**	93% *(477)*	7% *(35)*	43% *(225)*	57% *(294)*	9% *(48)*	69% *(354)*	22% *(112)*
Ethnicity							
**White**	93% *(1521)*	7% *(115)*	47% *(790)*	53% *(877)*	13% *(212)*	64% *(1057)*	23% *(378)*
**BAME**	85% *(80)*	15% *(14)*	44% *(41)*	56% *(52)*	17% *(15)*	53% *(47)*	30% *(27)*
Dog ownership							
**Don't own a dog/s**	92% *(1443)*	8% *(119)*	47% *(749)*	53% *(840)*	16% *(250)*	64% *(1003)*	20% *(314)*
**Own a dog/s**	94% *(571)*	7% *(40)*	55% *(338)*	46% *(282)*	12% *(74)*	61% *(372)*	27% *(166)*

For peer review only

Supplementary Table 3: Change in green space experience since restrictions by individual demographic and socio-economic characteristics (weighted).

	**Using green spaces benefits my mental health more now**		**Miss social interaction in green spaces now**		**Do more physical activity in green spaces now**	
	**Agree (N=689; 65%)**	**Disagree/neither agree nor disagree (N=368; 35%)**	**Agree (N=566; 54%)**	**Disagree/neither agree nor disagree (N=490; 46%)**	**Agree (N=309; 29%)**	**Disagree/neither agree nor disagree (N=746; 71%)**
	% *(N)*	% *(N)*	% *(N)*	% *(N)*	% *(N)*	% *(N)*
Sex						
**Male**	59% *(301)*	41% *(211)*	46% *(238)*	54% *(278)*	27% *(138)*	73% *(375)*
**Female**	71% *(387)*	29% *(157)*	61% *(328)*	39% *(211)*	32% *(171)*	69% *(371)*
Social grade						
**ABC1**	69% *(452)*	31% *(206)*	56% *(365)*	44% *(292)*	32% *(208)*	68% *(447)*
**C2DE**	59% *(237)*	41% *(162)*	51% *(201)*	50% *(197)*	25% *(101)*	75% *(299)*
Age						
**18-24**	60% *(64)*	40% *(42)*	61% *(66)*	39% *(42)*	46% *(50)*	55% *(60)*
**25-64**	69% *(500)*	31% *(229)*	53% *(383)*	47% *(342)*	30% *(216)*	70% *(508)*
**65+**	56% *(125)*	44% *(97)*	52% *(116)*	48% *(106)*	20% *(43)*	81% *(178)*
Ethnicity						
**White**	63% *(481)*	37% *(286)*	51% *(390)*	49% *(374)*	26% *(202)*	74% *(566)*
**BAME**	73% *(29)*	28% *(11)*	51% *(21)*	49% *(20)*	44% *(17)*	56% *(22)*
Dog ownership						
**Don't own a dog/s**	68% *(498)*	32% *(232)*	52% *(379)*	48% *(351)*	34% *(248)*	66% *(479)*
**Own a dog/s**	58% *(190)*	42% *(136)*	57% *(186)*	43% *(139)*	19% *(61)*	81% *(267)*

For peer review only

STROBE Statement—Checklist of items that should be included in reports of ***cross-sectional studies***

	Item No	Recommendation	Page No
**Title and abstract**	1	(*a*) Indicate the study's design with a commonly used term in the title or the abstract	P1
		(*b*) Provide in the abstract an informative and balanced summary of what was done and what was found	P2
**Introduction**			
Background/rationale	2	Explain the scientific background and rationale for the investigation being reported	P3
Objectives	3	State specific objectives, including any prespecified hypotheses	P3
**Methods**			
Study design	4	Present key elements of study design early in the paper	P4
Setting	5	Describe the setting, locations, and relevant dates, including periods of recruitment, exposure, follow-up, and data collection	P4
Participants	6	(*a*) Give the eligibility criteria, and the sources and methods of selection of participants	P4
Variables	7	Clearly define all outcomes, exposures, predictors, potential confounders, and effect modifiers. Give diagnostic criteria, if applicable	P4/5
Data sources/ measurement	8*	For each variable of interest, give sources of data and details of methods of assessment (measurement). Describe comparability of assessment methods if there is more than one group	P4
Bias	9	Describe any efforts to address potential sources of bias	P4, 6
Study size	10	Explain how the study size was arrived at	P4
Quantitative variables	11	Explain how quantitative variables were handled in the analyses. If applicable, describe which groupings were chosen and why	P5/6
Statistical methods	12	(*a*) Describe all statistical methods, including those used to control for confounding	P5/6
		(*b*) Describe any methods used to examine subgroups and interactions	P6
		(*c*) Explain how missing data were addressed	P4, 6
		(*d*) If applicable, describe analytical methods taking account of sampling strategy	P4, 6
		(*e*) Describe any sensitivity analyses	-
**Results**			
Participants	13*	(a) Report numbers of individuals at each stage of study—eg numbers potentially eligible, examined for eligibility, confirmed eligible, included in the study, completing follow-up, and analysed	Supplementary Tables 1-3
		(b) Give reasons for non-participation at each stage	-
		(c) Consider use of a flow diagram	-

For peer review only

Descriptive data	14*	(a) Give characteristics of study participants (eg demographic, clinical, social) and information on exposures and potential confounders	P5/6, Supplementary Table 1
		(b) Indicate number of participants with missing data for each variable of interest	Supplementary Table 1
Outcome data	15*	Report numbers of outcome events or summary measures	Tables 2 and 3 + Supplementary Tables 2 and 3
Main results	16	(*a*) Give unadjusted estimates and, if applicable, confounder-adjusted estimates and their precision (eg, 95% confidence interval). Make clear which confounders were adjusted for and why they were included	P6-9
		(*b*) Report category boundaries when continuous variables were categorized	-
		(*c*) If relevant, consider translating estimates of relative risk into absolute risk for a meaningful time period	-
Other analyses	17	Report other analyses done—eg analyses of subgroups and interactions, and sensitivity analyses	P7
**Discussion**			
Key results	18	Summarise key results with reference to study objectives	P9
Limitations	19	Discuss limitations of the study, taking into account sources of potential bias or imprecision. Discuss both direction and magnitude of any potential bias	P12
Interpretation	20	Give a cautious overall interpretation of results considering objectives, limitations, multiplicity of analyses, results from similar studies, and other relevant evidence	P9, 11, 12
Generalisability	21	Discuss the generalisability (external validity) of the study results	P12
**Other information**			
Funding	22	Give the source of funding and the role of the funders for the present study and, if applicable, for the original study on which the present article is based	P13

*Give information separately for exposed and unexposed groups.

Note: An Explanation and Elaboration article discusses each checklist item and gives methodological background and published examples of transparent reporting. The STROBE checklist is best used in conjunction with this article (freely available on the Web sites of PLoS Medicine at http://www.plosmedicine.org/, Annals of Internal Medicine at http://www.annals.org/, and Epidemiology at http://www.epidem.com/). Information on the STROBE Initiative is available at www.strobe-statement.org.

For peer review only - http://bmjopen.bmj.com/site/about/guidelines.xhtml

For peer review only

BMJ Open

Change in time spent visiting and experiences of green space following restrictions on movement during the COVID-19 pandemic: A nationally representative cross-sectional study of UK adults.

Journal:	*BMJ Open*
Manuscript ID	bmjopen-2020-044067.R1
Article Type:	Original research
Date Submitted by the Author:	02-Nov-2020
Complete List of Authors:	Burnett, Hannah; University of Glasgow, MRC/CSO Social and Public Health Sciences Unit<br>Olsen, Jonathan; University of Glasgow, MRC/CSO Social and Public Health Sciences Unit<br>Nicholls, Natalie; University of Glasgow, MRC/CSO Social and Public Health Sciences Unit<br>Mitchell, Richard; University of Glasgow, MRC/CSO Social and Public Health Sciences Unit
**Primary Subject Heading**:	Public health
Secondary Subject Heading:	Mental health
Keywords:	MENTAL HEALTH, COVID-19, PUBLIC HEALTH

SCHOLARONE™
Manuscripts

BMJ

I, the Submitting Author has the right to grant and does grant on behalf of all authors of the Work (as defined in the below author licence), an exclusive licence and/or a non-exclusive licence for contributions from authors who are: i) UK Crown employees; ii) where BMJ has agreed a CC-BY licence shall apply, and/or iii) in accordance with the terms applicable for US Federal Government officers or employees acting as part of their official duties; on a worldwide, perpetual, irrevocable, royalty-free basis to BMJ Publishing Group Ltd ("BMJ") its licensees and where the relevant Journal is co-owned by BMJ to the co-owners of the Journal, to publish the Work in this journal and any other BMJ products and to exploit all rights, as set out in our licence.

The Submitting Author accepts and understands that any supply made under these terms is made by BMJ to the Submitting Author unless you are acting as an employee on behalf of your employer or a postgraduate student of an affiliated institution which is paying any applicable article publishing charge ("APC") for Open Access articles. Where the Submitting Author wishes to make the Work available on an Open Access basis (and intends to pay the relevant APC), the terms of reuse of such Open Access shall be governed by a Creative Commons licence – details of these licences and which Creative Commons licence will apply to this Work are set out in our licence referred to above.

Other than as permitted in any relevant BMJ Author's Self Archiving Policies, I confirm this Work has not been accepted for publication elsewhere, is not being considered for publication elsewhere and does not duplicate material already published. I confirm all authors consent to publication of this Work and authorise the granting of this licence.

For peer review only

Change in time spent visiting and experiences of green space following restrictions on movement during the COVID-19 pandemic: A nationally representative cross-sectional study of UK adults.

Hannah Burnett[1], Jonathan R Olsen[1], Natalie Nicholls[1], Richard Mitchell[1]
[1]MRC/CSO Social and Public Health Sciences Unit, University of Glasgow, Berkeley Square, 99 Berkeley Street, Glasgow, G3 7HR, United Kingdom.

Correspondence:
Hannah Burnett
MRC/CSO Social and Public Health Sciences Unit,
University of Glasgow,
Berkeley Square,
99 Berkeley Street,
Glasgow,
G3 7HR.
Email: h.burnett.1@research.gla.ac.uk

Key words: mental health; COVID-19; public health.

Word Count: 4,000
Tables : 4
Figures : 1
Supplementary tables: 5

ABSTRACT

Objectives Green space positively influences health and well-being, however inequalities in use of green space are prevalent. Movement restrictions enforced due to the COVID-19 pandemic could have exacerbated existing inequalities regarding who visits green space. Therefore, this study aimed to explore how movement restrictions have changed the time spent visiting green space and experience of green space in the United Kingdom (UK) and how these differed by individual-level demographic characteristics.

Design and outcome measures A nationally representative cross-sectional survey administered through YouGov between 30th April–1st May 2020. Data were collected on the time spent visiting green space and change in the experience of green space, including missing social interaction, increased physical activity, and feeling greater mental health benefits in green space. Demographic information was collected on sex, age, ethnicity, social grade and dog ownership. Associations between specific outcome variables and predictors were assessed using logistic regression.

Setting United Kingdom, with population weights applied.

Participants 2,252 adults aged 18 years and over.

Results Overall, 63% of respondents reported a decrease in time spent visiting green space following movement restrictions. Lower social grade respondents were less likely to visit green space before *and* after restrictions were enforced (OR:0.35 (95% CI:0.24-0.51); OR:0.77 (95% CI:0.63-0.95)). Female respondents were more likely than male respondents to agree that green space benefitted their mental health more following restrictions (PP:0.70 vs. 0.59). Older (65+) respondents were less likely than middle age (25-64 years) respondents to have visited green space following the restrictions (OR:0.79 (95% CI:0.63-0.98)).

Conclusions Inequalities in green space use were sustained, and possibly exacerbated, during movement restrictions. Our findings emphasise the importance of green spaces remaining open globally in any future 'lockdowns'/pandemics. Further investigation is required to determine how visit patterns and experiences change through the different stages of the COVID-19 pandemic in the UK.

(Word Count: 300)

Strengths and Limitations of this study

- Our data are currently the only existing data covering change in the time spent visiting green space and experiences of green space for the UK population following the movement restrictions enforced due to the COVID-19 pandemic.
- The sample is nationally representative of UK adults.
- Collecting data on *both* time spent visiting green space and change in experience of green space during the movement restrictions is a strength of this study, compared to other surveys exploring change in green space use during the COVID-19 pandemic, including the Google COVID-19 Community Mobility Reports and Natural England's People and Nature Survey.

For peer review only - http://bmjopen.bmj.com/site/about/guidelines.xhtml

- The data collected for this study were from a cross-sectional survey, therefore it is not possible to demonstrate causality between change in green space use and experiences and the demographic data.
- Certain variable response categories were necessarily recategorised for analysis, which meant that some distinctiveness across groups were lost, particularly for the ethnicity and age variables.

INTRODUCTION

Evidence suggests that exposure to green space has a positive influence on health and well-being.[1,2] Green space use is associated with increased levels of social interaction and physical activity, and decreased levels of all-cause mortality, loneliness and stress.[3–5] Additionally, there is evidence that contact with green space may disproportionately benefit disadvantaged populations, reducing health inequalities and therefore weakening the effects of poverty – known as the ‘equigenic’ effect.[6,7]

The health impacts of contact with green spaces are quite well studied by both observational and experimental designs. There is rather less literature on the levels of, motivations for, and barriers to visiting green spaces in the first place. Recent surveys by Natural England and Scottish Natural Heritage have found that 74% of the English population and 70% of the Scottish population visit green space ‘frequently’ (once a month or more often). In both countries, the top three reasons stated for frequent green space use were health/exercise, dog walking, and to relax/unwind.[8,9] Further research exploring the motivations and reasons for visiting green space found that visitation frequency is affected by an individual’s feelings of nature connectedness, as well as their childhood exposure to nature.[10–12]. It is important to note that substantial inequalities in green space use have also been reported, with studies finding that females, older people and those from less advantaged socioeconomic positions (SEP) are more likely to be infrequent users than their male, younger and higher SEP counterparts.[9,13–15]

In 2020, the United Kingdom (UK) experienced major disruption to everyday life due to the novel coronavirus disease 2019 (COVID-19) pandemic. COVID-19 is an infectious disease first identified in the city of Wuhan, China in December 2019.[16] As a response to the COVID-19 outbreak, the UK announced a series of movement restrictions from 23rd March 2020. These included rules designed to increase social distancing, meaning that people could only leave their households to make ‘essential trips’ for food, medication and exercise.[17] Recent studies have highlighted the negative effects of COVID-19 on the UK population’s mental health and wellbeing, which are likely to be profound and long-lasting.[18] Research exploring the wider health effects of COVID-19 suggest that the negative indirect effects are being borne disproportionately by people who have fewer resources and poorer health.[19] If natural environments usually act to mitigate the connections between adversity and poor health,[6,7] it is important to assess the extent to which lockdown affected both use and experience of such environments. Future lockdowns and movement restrictions are highly likely as second, third and perhaps fourth waves of the pandemic take place around the world.

The aim of this research was to explore changes in the time spent visiting green space and experience of visits to green space among the UK population following movement restrictions. The sociodemographic characteristics focused on were those identified by the literature as being most consistent markers of inequality in use. The research questions were:

1) How did time spent visiting green space change following movement restrictions, compared with before, and for whom?
2) How did experiences of visits to green space change following the restrictions, and did any change vary by sex, social grade, age, ethnicity and dog ownership? Specifically,
   a) Did respondents feel that green space benefitted their mental health more since restrictions than before?
   b) Did respondents miss social interaction in green space more following movement restrictions?
   c) Had physical activity increased following movement restrictions?

METHODS

Survey design and sample

An online cross-sectional survey was administered by YouGov between 30th April and 1st May 2020.[20] Questions were answered by a sample of 2,252 adults from the UK (aged 18 and over). The sample was drawn from a panel of over 800,000 individuals who specifically opted in to participate in online research activities. Sample members were randomly selected at random from the panel and sent an email providing a survey link. Table 1 shows the themes and specific survey instruments analysed in this paper. Only the respondents that visited green space following movement restrictions were asked the questions regarding change in experience of green space. Demographic information about participants was also collected (including sex, age and social grade, which was classified by occupation).[21,22] Although the sample was reasonably large, small numbers in variable response categories necessitated some category mergers (Table 1 and Supplementary Table 1).

The survey covered adults from across the UK, with respondents from England (N=1,875), Scotland (N=209), Wales (N=107), and Northern Ireland (N=61). Weightings were applied to render the sample representative of UK adults (detailed below). When the survey was distributed, the same movement restrictions were implemented across the UK. These included only leaving home for limited purposes, such as medical needs, shopping for basic necessities (food and medicine), and exercising once a day alone/with members of your household.[23] Since then, the individual parliaments/assemblies representing these countries have imposed different COVID-19 policies.

Table 1: Survey themes and specific items analysed, including variables recategorised.

Themes	Question/statement	Potential responses	Recategorised
Change in the amount of time spent visiting green space	*"Did you EVER visit a green space in the year before the movement restrictions were enforced in the UK?"*	Yes, I did<br>No, I didn't<br>Don't know/ can't recall	-<br><br>*Don't know/can't recall excluded (N=80, 3.5%)*
	"Please think about your behaviour since the UK enforced a 'lock down' to restrict movement, as a result of the current Coronavirus (COVID-19) outbreak (i.e. since 23rd March 2020). Have you visited a green space since the movement	Yes, I have<br>No, I haven't<br>Don't know/ can't recall	-

	restrictions have been enforced in the UK?"		*Don't know/can't recall excluded (N=43, 1.9%)*
	"How much, if at all, has the amount of time that you have spent visiting green spaces changed since the 'lock down' movement restrictions began (i.e. 23rd March 2020) compared to before?"	Increased a lot<br>Increased a little<br>No difference<br>Decreased a little<br>Decreased a lot<br>Don't know	'Increased' (increased a lot and increased a little)<br>'Same' (no difference)<br>'Decreased' (decreased a lot and decreased a little)<br><br>*Don't know excluded (N=74, 3.3%)*
Experience change (If respondent had visited a green space since the movement restrictions were enforced)	*"I feel that being in green spaces benefits my mental health more now, than before the movement restrictions were in place"*	Strongly agree<br>Slightly agree<br>Neither agree nor disagree<br>Slightly disagree<br>Strongly disagree<br>Don't know/ can't recall	'Agree' (strongly agree and slightly agree)<br>'Neither' (neither agree nor disagree)<br>'Disagree' (slightly disagree and strongly disagree)<br><br>*Don't know/can't recall excluded (N=7, 0.6%)*
	"I have missed seeing and/ or talking with people in green spaces since the movement restrictions were introduced"	Strongly agree<br>Slightly agree<br>Neither agree nor disagree<br>Slightly disagree<br>Strongly disagree<br>Don't know/ can't recall	'Agree' (strongly agree and slightly agree)<br>'Neither' (Neither agree nor disagree)<br>'Disagree' (slightly disagree and strongly disagree)<br><br>*Don't know/can't recall excluded (N=8, 0.8%)*
	"I do more physical activity in green spaces now, than I did before the movement restrictions were introduced"	Strongly agree<br>Slightly agree<br>Neither agree nor disagree<br>Slightly disagree<br>Strongly disagree<br>Don't know/ can't recall	'Agree' (strongly agree and slightly agree)<br>'Neither' (Neither agree nor disagree)<br>'Disagree' (slightly disagree and strongly disagree)<br><br>*Don't know/can't recall excluded (N=9, 0.8%)*

Don't know/ can't recall responses were all excluded from analysis (weighted counts and proportions reported above).

Demographic variables

Individual level demographic and socio-economic characteristics were captured from the survey, as follows: sex (male, female); age group (18-24 years, 25-64 years, 65+ years); ethnicity (White, Black, Asian and minority ethnic (BAME)); dog ownership (Yes, No); and social grade (ABC1, C2DE), derived by YouGov from combined occupational social grade categories. ABC1 was the higher social grade, and included non-manual workers, for example, senior managers and owners of small establishments. C2DE was the lower social grade, and included all manual workers, for example, shop assistants and labourers.[24] Hereafter, social grade will be described as higher and lower social grade.

Patient and Public Involvement

There was no direct patient or public involvement in this study.

For peer review only - http://bmjopen.bmj.com/site/about/guidelines.xhtml

Analyses

Descriptive statistics

The count and proportion of respondents who had: visited green space before and after movement restrictions were enforced; increased or decreased visitation; and agreed or disagreed with the three change in experience statements (Table 1) are presented. These were also explored by sex, age, ethnicity, dog ownership and social grade.

Statistical analysis

Binary logistic regression analyses were conducted to assess the association between the individual predictor variables and the following two outcomes:

1. having visited green space in the year before movement restrictions were enforced;
2. having visited green space since movement restrictions were enforced, with this analysis being restricted to those who had reported visiting green space before movement restrictions.

The associations between the individual predictor variables and each of the following outcomes were also assessed using multinomial logistic regression or, if appropriate, ordinal logistic regression, with results presented as predicted probabilities (PP):

1. Change in green space visitation following movement restrictions;
2. Levels of agreement that green space benefits their mental health more now (since movement restrictions compared to before);
3. Levels of agreement that they miss seeing/talking to people in green space now (since movement restrictions compared to before); and
4. Levels of agreement that they do more physical activity in green space now (since movement restrictions compared to before).

The results of the binary logistic regression analyses were expressed as odds ratios (OR) with 95% confidence intervals (CIs). Other results are presented as predicted outcome group probabilities for each variable. Univariate models for each predictor were conducted first, followed by a fully adjusted model containing all predictors for each outcome. Weightings were calculated by YouGov, with the final data weighted to match the national profile of all adults aged 18 and over and applied during analyses to render the sample representative of UK adults.[22] All analyses were conducted in R v3.5.1,[25], and the *brant* package,[26], was used to check that the proportionality of odds assumption for ordinal logistic regression was not violated.[27] A full R script is available on GitHub.[28]

Interactions

Given existing literature,[9,13,29,30] we expected some interactions between the demographic variables sex, age, social grade, and ethnicity, in their relationships with change in visit time and experience of green space following movement restrictions. We therefore explored interactions between each of these demographic variables for every model, assessing their significance via Wald tests, and then producing predicted probabilities to aid interpretation of the significant interactions.

For peer review only - http://bmjopen.bmj.com/site/about/guidelines.xhtml

RESULTS

Descriptive statistics

93% of all respondents had visited green space in the year before movement restrictions were enforced. In contrast, 53% of respondents reported visiting green space following movement restrictions. 63% of respondents reported that the amount of time they spent in green space had decreased since movement restrictions compared to before, with 15% reporting an increase, and 22% reporting no difference in the time spent visiting green space (Supplementary Table 2).

A greater proportion of respondents agreed (65%) than disagreed (10%) or neither agreed nor disagreed (25%) that green space benefited their mental health more following movement restrictions compared to before. More respondents agreed (54%) that they missed social interaction in green space more since movement restrictions, than disagreed (19%) or neither agreed nor disagreed (27%). 39% of respondents disagreed that they had increased physical activity in green space since movement restrictions, compared to 29% who agreed and 32% that neither agreed nor disagreed (Supplementary Table 3).

Change in visitation time

Visiting green space before movement restrictions

In the adjusted logistic regression model for visiting green space in the year before movement restrictions were enforced (Table 2), only two variables had significant associations ($p<0.05$). Lower social grade respondents (OR:0.35 (95%CI:0.24-0.51)) and BAME (OR:0.43 (95%CI:0.23-0.80)) respondents had lower odds of visiting green space before movement restrictions, compared to higher social grade and white respondents.

Visiting green space following movement restrictions

Respondents in the lower social grade group were less likely to have visited green space than respondents in the higher social grade group (OR:0.77 (95%CI:0.63-0.95)) (Table 2). Older respondents (65+) were also less likely than middle age respondents (25-64) to have visited green space following the movement restrictions (OR:0.79 (95% CI:0.63-0.98)). Respondents who owned a dog/s were more likely than respondents who did not to have visited green space after movement restrictions were enforced (OR:1.42 (95%CI:1.14-1.78)).

Change in time spent visiting green space

Lower social grade respondents were less likely (had a lower predicted probability) than higher social grade respondents to report increased visits following movement restrictions (PP:0.09 vs. 0.16)(Table 3). Older respondents (aged 65+) were the least likely to report increased green space visitations (PP:0.09(65+) vs. 0.14(25-64) and 0.21(18-24)). Sex was significantly associated with *decreased* visits. Females were more likely (had a higher predicted probability) to report decreased green space visits compared to males (PP:0.67 vs. 0.62)(Table 3). Respondents without a dog/s were more likely to report decreased visitations to green space compared to dog owners (PP:0.66 vs. 0.62).

For peer review only - http://bmjopen.bmj.com/site/about/guidelines.xhtml

Interaction effects

Six interactions were significant ($p<0.05$); one between social grade and ethnicity in their association with visiting green space before movement restrictions. Two between age and ethnicity, in their association with visiting green spaces since movement restrictions and with *increased* visits since movement restrictions. The three experience outcomes (mental health benefits, miss social interaction and increased physical activity) had significant interactions between social grade and age.

Higher social grade white respondents had the highest probability of visiting green space before restrictions, but lower social grade BAME respondents were more likely to have visited green space before restrictions than the higher social grade BAME respondents (Supplementary Table 4). Young BAME respondents had a higher probability of visiting green space and reporting increased visits following restrictions compared to white and older BAME respondents (Figure 1). Older BAME and white respondents had a higher probability of reporting decreased visits following movement restrictions than their corresponding middle age respondents. However, the number of BAME respondents, especially young and older BAME respondents, in this sample was small and these results should be interpreted with caution.

For the interactions associated with the experience outcomes, younger respondents from the higher social grade group had the highest probability of agreeing both that green space benefitted their mental health more, and that they missed social interaction in green space, following the movement restrictions. In contrast, younger respondents in the lower social grade group had the highest probability of disagreeing that mental health benefitted their mental health more. Older respondents in the lower social grade group had a higher probability of agreeing that they missed social interaction in green space than younger respondents. Finally, younger respondents had the highest probability of agreeing that they had increased physical activity following the movement restrictions compared to the older respondents in both social grade groups (Supplementary Table 5).

For peer review only - http://bmjopen.bmj.com/site/about/guidelines.xhtml

Table 2: Adjusted binary logistic regression models predicting green space visit before and after the movement restrictions were enforced. OR = odds ratio; CI = confidence intervals; *p<0.05*.

	**Visited green space in the year before restrictions (yes)**		**Visited green space after restrictions (yes & visited green space before restrictions)**	
	Adjusted		Adjusted	
	OR (95% CI)	p-value	OR (95% CI)	p-value
Sex				
**Male (ref)**				
**Female**	1.35 (0.94-1.95)	0.109	0.88 (0.72-1.07)	0.205
Social grade				
**ABC1 (ref)**				
**C2DE**	0.35 (0.24-0.51)	*<0.001*	0.77 (0.63-0.95)	*0.013*
Age				
**18-24**	2.92 (1.00-8.57)	0.051	0.81 (0.54-1.21)	0.297
**25-64 (ref)**				
**65+**	1.22 (0.80-1.85)	0.353	0.79 (0.63-0.98)	*0.035*
Ethnicity				
**White (ref)**				
**BAME**	0.43 (0.23-0.80)	*0.007*	1.06 (0.67-1.68)	0.799
Dog ownership				
**No (ref)**				
**Yes**	1.29 (0.83-2.00)	0.260	1.42 (1.14-1.78)	*0.002*

For peer review only - http://bmjopen.bmj.com/site/about/guidelines.xhtml

Table 3: Multinomial logistic regression models predicting change in time visiting green space and levels of agreement that green space benefits their mental health more now (since movement restrictions were enforced compared to before). **Significant predicted probabilities**; *p<0.05.*

		**Change in time spent visiting green space**				**Using green spaces benefits my mental health more now**			
		**Decreased**	**Same**	**Increased**	**p-value**	**Agree**	**Neither**	**Disagree**	**p-value**
Sex	**Male**	**0.62**	0.25	0.13	*0.041*	**0.59**	0.31	0.10	*0.004*
	**Female**	**0.67**	0.20	0.12		**0.70**	0.22	0.09	
Social grade	**ABC1**	**0.65**	0.19	**0.16**	*<0.001*	**0.68**	0.24	0.09	*0.048*
	**C2DE**	**0.64**	0.27	**0.09**		**0.59**	0.31	0.10	
Age	**18-24**	0.57	0.22	0.21	*0.004*	0.55	0.20	**0.25**	*<0.001*
	**25-64**	0.63	0.22	0.14		0.68	0.22	0.10	
	**65+**	0.69	0.22	**0.09**		**0.55**	0.38	**0.06**	
Ethnicity	**White**	0.22	0.65	0.12	0.167	0.64	0.26	0.10	0.063
	**BAME**	0.30	0.56	0.14		0.73	0.25	0.02	
Dog ownership	**No**	**0.66**	0.21	**0.13**	*0.003*	0.67	0.24	0.09	0.117
	**Yes**	**0.62**	0.28	**0.10**		0.59	0.30	0.11	

Table 4: Multinomial logistic regression models predicting levels of agreement that respondents missed seeing/talking to people in green space more since movement restrictions were enforced compared to before, and levels of agreement that respondents do more physical activity in green space following the movement restrictions. **Significant predicted probabilities**; $p<0.05$.

		Miss social interaction in green spaces now				Do more physical activity in green spaces now			
		**Agree**	**Neither**	**Disagree**	**p-value**	**Agree**	**Neither**	**Disagree**	**p-value**
Sex	**Male**	0.45	0.30	0.25	*<0.001*	0.24	0.37	0.40	0.096
	**Female**	**0.58**	0.26	0.16		0.27	0.29	0.43	
Social grade	**ABC1**	0.53	0.27	0.20	0.256	0.27	0.31	0.42	0.307
	**C2DE**	0.48	0.31	0.21		0.23	0.36	0.41	
Age	**18-24**	0.59	0.23	0.18	0.672	0.44	0.23	0.33	*0.002*
	**25-64**	0.51	0.28	0.21		0.29	0.31	0.40	
	**65+**	0.53	0.30	0.18		**0.18**	0.38	0.45	
Ethnicity	**White**	0.52	0.28	0.20	0.802	0.25	0.32	0.42	0.063
	**BAME**	0.52	0.31	0.16		0.33	0.44	**0.22**	
Dog Ownership	**No**	0.50	0.30	0.20	0.295	0.31	0.30	0.39	*<0.001*
	**Yes**	0.55	0.25	0.20		**0.17**	0.38	0.46	

Change in visit experience

Mental health benefits

Females were more likely to agree than males that being in green space benefitted their mental health more following movement restrictions than before (PP:0.70 vs. 0.59). Higher social grade respondents were more likely to agree than lower social grade respondents (PP:0.68 vs. 0.59). Younger respondents were more likely to disagree that being in green space benefitted their mental health more following movement restrictions than before, whilst older respondents were less likely to disagree (PP:0.25(18-24) vs. 0.06(65+) and 0.10(25-64))(Table 3).

Missed social interaction

Female respondents were more likely to agree that they missed seeing and talking with other people in green space since movement restrictions compared to before than male respondents (PP:0.58 vs. 0.45)(Table 4).

Increased physical activity

Older respondents were less likely to agree that they had increased physical activity following movement restrictions (PP:0.18(65+) vs. 0.20(25-64) and 0.44(18-24)). Respondents who owned a dog/s were less likely than respondents without a dog/s to agree (PP:0.17 vs. 0.31)(Table 4).

DISCUSSION

Our findings suggest that inequalities in use of green space between demographic groups were sustained following movement restrictions, with lower social grade individuals less likely than higher social grade individuals to have visited green space before and since movement restrictions. Other existing inequalities in use were possibly exacerbated in the month after movement restrictions were enforced, with females being more likely to report a decrease in visits following movement restrictions.

The proportion of respondents who visited green space before and following movement restrictions decreased, from 93% to 53%. This was consistent with Natural England's findings from April 2020 where 49% of English adults reported green space visits in the previous two weeks.[31] Natural England conducted an online panel survey in April 2020 (N=2,083), the survey covered only the English population, collecting information on the frequency of time in green and natural spaces in the 12 months, and then 2 weeks, prior to the survey.[32] The survey did not directly ask respondents about whether they felt their experiences within green space had changed following movement restrictions. A similar study focused on change in time spent visiting parks using the Google COVID-19 Community Mobility Reports covering 620 counties across the United States(USA). They found a lower percentage decrease in park visits compared to our findings, reporting a 17-35% decrease in visits between 15th March-9th May 2020.[33] This difference may be explained by the focus on parks alone rather than different types of green space. However, additional research exploring the Google COVID-19 Community Mobility Reports found that

For peer review only - http://bmjopen.bmj.com/site/about/guidelines.xhtml

from 16th February-29th March 2020 park use decreased by 90% in Catalonia, 7% in Oslo, and 79% in New York County. In Stockholm, park use increased by 24% in the same timeframe.[34] This could be explained by Sweden having a less stringent approach to movement restrictions and instead relying on 'self-responsibility' to prevent the population having to restrict movement and stay at home.[35] The Swedish population may also be more culturally attuned to seeking time in nature to combat stress.

Females and older individuals were more likely to have reported decreased visits to green space following movement restrictions. These findings corroborate with existing data collected before movement restrictions.[9,13,14] Boyd *et al.* analysed the Natural England 'Monitor of Engagement with the Natural Environment' survey (N=63,890) with a focus on infrequent use. They found that females and older adults in England were more likely to be infrequent visitors.[9] Cohen *et al.* explored physical activity levels in Los Angeles' parks (N=1,318). They reported that age and sex were predictors of park use, with <5% of park users over 60 years old, and males using parks more often than females (62% vs. 38%).[13]

Our findings suggested that existing patterns of gender inequality in use were sustained and potentially exacerbated due to fears arising from the COVID-19 pandemic. For example, an Ipsos MORI survey was conducted in April 2020, collecting data on gender differences in British attitudes towards COVID-19. They found that women were more likely to report following government rules to avoid leaving their home (78%) than men (68%) and felt more uncomfortable about returning to "normal"(N=1,000).[36,37]

Previous studies have found that females feel more vulnerable than males in green spaces, especially without company.[14,15,29,38–40] There are few quantitative studies focusing on sex and the importance of social interaction as a motivation behind green space use. We found that females were more likely to agree that they missed seeing/talking with others in green space than males. This could be explained by the nature of the movement restrictions at this time, particularly the inability to socially interact with individuals outside your household in green space.

Given older age is probably the most important risk factor for an adverse outcome from COVID-19,[41,42] it was not surprising that older individuals were less likely to report an increase in green space visits and to agree that they were doing more physical activity following movement restrictions. These findings corroborate with the majority of research exploring older age and green space use before the movement restrictions.[9,13,43] Older people in the UK have been found to be at particular risk of social isolation during social distancing, being less likely to use online communications and more likely to live alone than younger individuals.[19] Our findings also show that older people are less likely to have used green space during lockdown, further emphasising this point. The significant decrease in green space visits following movement restrictions for older respondents could be explained by the government advice for over 70s and those with an underlying health condition to shield, minimise interaction and stay at home, for around 12 weeks from 21st March 2020.[44]

Lower social grade respondents reported little or no change in visitation to green space, with time spent visiting green space remaining low both before and after movement restrictions. This is supported by previous studies exploring the association between socio-economic position (SEP) and green space use.[9,15,43] One possible explanation is a lack of interest in visiting green space reported by lower SEP individuals.[9] Additionally, the social grade

For peer review only - http://bmjopen.bmj.com/site/about/guidelines.xhtml

variable was categorised by occupation, and it was likely that individuals categorised as lower social grade were working in manual or service occupations.[24] They may have continued at their usual workplace/working hours during the COVID-19 pandemic. Findings from the Office for National Statistics support this explanation, with 30.5% of employees in the bottom three income deciles (monthly earnings of up to £1,450) considered as key workers in March/April 2020, compared to 26.4% in the top three income deciles (monthly earnings of up to £3,250).[45] Similarly, existing international research on public space use during COVID-19 restrictions worldwide state that skilled workers in the knowledge economy have shifted easily into online work from home, and can therefore make more use of green spaces during the movement restrictions.[46]

Younger BAME respondents had a higher probability of visiting green space following movement restrictions and reporting increased visits since movement restrictions, compared to white and older BAME respondents. A survey on park use and ethnic integration in Reading, UK (N=294) reported similar findings, with Black and Asian youths accounting for a higher proportion of parks users than white youths.[47] Further exploration is required to enable interpretation of these interaction results because of our small BAME sample.

The results for dog owners suggest that the movement restrictions have had an overall negative impact on their experiences within green space. Although dog owners were more likely to have visited green space following the movement restrictions than those that did not own a dog, they were less likely to agree that they had increased physical activity and more likely to have decreased visitations following the restrictions. These results differ from research undertaken in Canada that reported findings that dog ownership was associated with more outdoor play and less indoor play in Canadian youth at the start of the pandemic.[48] However, the focus on youth rather than adults may explain this difference in findings. Instead, the difference found in visits after restrictions were enforced may be due to dog owners having to walk their dog/s in green spaces *despite* the pandemic restrictions. In comparison between our results and those of other studies, we are mindful of the difference between number of visits and time spent in green spaces as measures of 'use'. It would be entirely possible to increase one at the expense of the other and our data were not well suited to unpacking the relationship between them.

Strengths and limitations

Our study has several strengths; to our knowledge, currently this is the only data covering UK population change in use and experiences within green space at the start of the movement restrictions from 23rd March 2020. This means that the data could provide timely information to local and national governments across the UK. It is important to understand the effects of the initial movement restrictions to generate policy recommendations for any future movement restrictions. The rapid collection of data, just over a month after the movement restrictions were introduced, reduces risk of recall bias. The sample is nationally representative of UK adults, with weightings calculated by YouGov and applied to all analyses, reducing risk of selection bias.[22]

A further strength of our study is providing quantitative data on the importance of social interaction as a driver for using green space, which is under-reported in the literature. We analysed social grade at an individual level, which provides greater detail and accuracy of the respondent's SEP than at neighbourhood level.

For peer review only - http://bmjopen.bmj.com/site/about/guidelines.xhtml

There are some caveats. The survey design was cross-sectional, and we are unable to demonstrate causality between change in green space use and experiences and the demographic data. Additionally, the data on use and experiences are self-reported and therefore subjective. Certain variable response categories had to be recategorised in order to analyse them. This is particularly evident in the ethnicity and age variables, where some distinctiveness across groups was lost. The variable best capturing change in behaviour measured time spent in green space, but this made it difficult to understand whether respondents traded off time and numbers of visits.

We encourage future research to further investigate the interactions between ethnicity, age and social grade in relation to green space use to enable more substantial interpretation of the results and to explore whether the patterns are consistent over time and in different contexts. Moreover, further research is required to explore the reasons *why* many patterns and changes in use of green space since movement restrictions have emerged. It would also be interesting to explore the change in patterns of use before and following movement restrictions for those that did not visit before the movement restrictions but did visit green space following restrictions.

CONCLUSION

During the COVID-19 pandemic, our study provides novel evidence to suggest that green space use and experiences were affected during the first month of movement restrictions in the UK. Our findings suggested that inequalities in use were sustained, with lower social grade individuals less likely to visit green space than higher social grade individuals before and following movement restrictions. It is possible that these inequalities were exacerbated, as females and older individuals were the groups most likely to have decreased visits following movement restrictions. Although these findings reflect the UK population's experience during the movement restrictions, they could be compared with countries such as Norway, USA, and Spain where green space visits also decreased. We believe that these findings emphasise the need for green spaces to remain open in any future 'lockdowns' and for governments to actively encourage individuals to utilise these spaces to support their mental and physical health during subsequent waves of the pandemic. Further investigation is required to support these findings and how they may change through the stages of the COVID-19 pandemic and the relaxing, and potential re-introduction, of movement restrictions.

COMPETING INTERESTS

None declared.

DATA SHARING STATEMENT

We have made our research dataset publicly available (Datacite DOI: 10.5525/gla.researchdata.1038 - embargoed until 31/10/2023).

CONTRIBUTORS

HB, JRO and RM designed the study and survey; HB conducted data analysis which was reviewed by NN; HB wrote the first draft of the paper; all authors contributed to interpreting the results and revising the draft. The corresponding author attests that all listed authors meet authorship criteria and that no others meeting the criteria have been omitted.

FUNDING STATEMENT

For peer review only - http://bmjopen.bmj.com/site/about/guidelines.xhtml

The authors (HB, JRO, NN & RM) are part of the Places and Health Programme at the MRC/CSO Social and Public Health Sciences Unit (SPHSU), University of Glasgow, supported by the Medical Research Council (MC_UU_12017/10) and the Chief Scientist Office (SPHSU10).

References:

1 Barton J, Pretty J. What is the Best Dose of Nature and Green Exercise for Improving Mental Health? A Multi-Study Analysis. *Environ Sci Technol* 2010;**44**:3947–55. doi:10.1021/es903183r

2 Douglas O, Lennon M, Scott M. Green space benefits for health and well-being: A life-course approach for urban planning, design and management. *CITIES* 2017;**66**:53–62. doi:10.1016/j.cities.2017.03.011

3 Twohig-Bennett C, Jones A. The health benefits of the great outdoors: A systematic review and meta-analysis of greenspace exposure and health outcomes. *Environ Res* 2018;**166**:628–37. doi:10.1016/j.envres.2018.06.030

4 Maas J, Van Dillen SME, Verheij RA, *et al.* Social contacts as a possible mechanism behind the relation between green space and health. *Health Place* 2009;**15**:586–95. doi:10.1016/j.healthplace.2008.09.006

5 Wells N, Evans G. NEARBY NATURE A Buffer of Life Stress Among Rural Children. *Environ Behav* 2003;**35**:311–30. doi:10.1177/0013916503251445

6 Frumkin H, Bratman GN, Breslow SJ, *et al.* Nature Contact and Human Health: A Research Agenda. *Environ Health Perspect* 2017;**125**. doi:10.1289/EHP1663

7 Mitchell RJ, Richardson EA, Shortt NK, *et al.* Neighborhood Environments and Socioeconomic Inequalities in Mental Well-Being. *Am J Prev Med* 2015;**49**:80–4. doi:10.1016/j.amepre.2015.01.017

8 Wilson V, Seddon B. Scotland's People and Nature Survey 2017/18 – outdoor recreation and health modules. (Scottish Natural Heritage Research Report No. 1062). Inverness: 2018.

9 Boyd F, White MP, Bell SL, *et al.* Who doesn't visit natural environments for recreation and why: A population representative analysis of spatial, individual and temporal factors among adults in England. *Landsc Urban Plan* 2018;**175**:102–13. doi:10.1016/j.landurbplan.2018.03.016

10 Bell SL, Phoenix C, Lovell R, *et al.* Green space, health and wellbeing: making space for individual agency. *Health Place* 2014;**30**:287–92. doi:10.1016/j.healthplace.2014.10.005

11 Lin BB, Fuller RA, Bush R, *et al.* Opportunity or Orientation ? Who Uses Urban Parks and Why. 2014;**9**:1–7. doi:10.1371/journal.pone.0087422

12 Ward Thompson C, Aspinall P, Montarzino A. The Childhood Factor Adult Visits to Green Places and the Significance of Childhood Experience. *Environ Behav* 2008;**40**:111–43. doi:10.1177/0013916507300119

13 Cohen DA, McKenzie TL, Sehgal A, *et al.* Contribution of Public Parks to Physical Activity. *Am J Public Health* 2007;**97**:509–14. doi:10.2105/AJPH.2005.072447

14 Lee ACK, Maheswaran R. The health benefits of urban green spaces: A review of the evidence. *J Public Health (Bangkok)* 2011;**33**:212–22. doi:10.1093/pubmed/fdq068

15 Morris J, O'Brien E, Ambrose-Oji B, *et al.* Access for all? Barriers to accessing woodlands and forests in Britain. *Local Environ Int J Justice Sustain* 2011;**16**:375–96. doi:10.1080/13549839.2011.576662

16 Wynants L, Van Calster B, Bonten MMJ, *et al.* Prediction models for diagnosis and prognosis of covid-19 infection: systematic review and critical appraisal . *BMJ*

For peer review only - http://bmjopen.bmj.com/site/about/guidelines.xhtml

2020;**369**. doi:10.1136/bmj.m1328

17 Johnson B. Prime Minister's statement on coronavirus (COVID-19): 23 March 2020. GOV.UK. 2020.https://www.gov.uk/government/speeches/pm-address-to-the-nation-on-coronavirus-23-march-2020 (accessed 7 Jul 2020).

18 O'Connor RC, Wetherall K, Cleare S, *et al.* Mental health and wellbeing during the COVID-19 pandemic: longitudinal analyses of adults in the UK COVID-19 Mental Health & Wellbeing study. *Br J Psychiatry* 2020;:1–17. doi:10.1192/bjp.2020.212

19 Douglas M, Katikireddi SV, Taulbut M, *et al.* Mitigating the wider health effects of covid-19 pandemic response. *BMJ* 2020;**369**. doi:10.1136/bmj.m1557

20 YouGov. About YouGov Company. https://yougov.co.uk/about/ (accessed 8 Jun 2020).

21 YouGov and ESOMAR (World Association for Social Opinion and Market Research). ESOMAR 28: 28 Questions to help Online Research Buyers. The Netherlands: 2012. http://cdn.yougov.com/cumulus_uploads/document/t3r5k565j5/ESOMAR_28.pdf (accessed 8 Jun 2020).

22 YouGov. Panel Methodology. https://yougov.co.uk/about/panel-methodology/ (accessed 8 Jun 2020).

23 Cabinet Office. Staying at home and away from others (social distancing). London: 2020. https://www.gov.uk/government/publications/full-guidance-on-staying-at-home-and-away-from-others (accessed 8 Oct 2020).

24 The Market Research Society (MRS). Definitions employed in Social Grading. MRS. https://www.mrs.org.uk/pdf/Definitions used in Social Grading based on OG7.pdf (accessed 2 Jul 2020).

25 R Core Team. R: A Language and Environment for Statistical Computing. 2018.https://www.r-project.org/

26 Schlegel BE. Package brant. https://benjaminschlegel.ch/en/r/brant/ (accessed 29 Oct 2020).

27 Brant R. Assessing proportionality in the proportional odds model for ordinal logistic regression. *Biometrics* 1990;**46**:1171–8.

28 Burnett H. Change in green space use and experiences following UK restrictions on movement. GitHub. 2020.https://github.com/hannahburnett/greenspace-covid_code (accessed 21 Jul 2020).

29 Richardson EA, Mitchell R. Gender differences in relationships between urban green space and health in the United Kingdom. *Soc Sci Med* 2010;**71**:568–75. doi:10.1016/J.SOCSCIMED.2010.04.015

30 Astell-Burt T, Mitchell R, Hartig T, *et al.* The association between green space and mental health varies across the lifecourse. A longitudinal study. *J Epidemiol Community Heal* 2014;**68**:578–83. doi:10.1136/jech-2013-203767

31 Natural England. The People and Nature Survey for England: Monthly interim indicators for April 2020 (Experimental Statistics) . Nat. Engl. 2020.https://www.gov.uk/government/publications/the-people-and-nature-survey-for-england-monthly-interim-indicators-for-april-2020-experimental-statistics/the-people-and-nature-survey-for-england-monthly-interim-indicators-for-april-2020-experimental-statistics (accessed 25 Jun 2020).

32 Natural England. The People and Nature Survey For England. 2020. http://publications.naturalengland.org.uk/publication/6382837173583872

33 Curtis DS, Rigolon A, Schmalz DL, *et al.* Getting out while staying in: Park use decreased during the COVID-19 pandemic, especially where park availability was low. *Cent Open Sci OSF Prepr* Published Online First: 2020. doi:10.31235/osf.io/9xzgf

For peer review only - http://bmjopen.bmj.com/site/about/guidelines.xhtml

34 Barton D, Haase D, Mascarenhas A, *et al.* Enabling Access to Greenspace During the Covid-19 Pandemic-Perspectives from Five Cities. Nat. Cities. 2020.https://www.thenatureofcities.com/2020/05/04/enabling-access-to-greenspace-during-the-covid-19-pandemic-perspectives-from-five-cities/ (accessed 23 Oct 2020).

35 Savage M. Lockdown, what lockdown? Sweden's unusual response to coronavirus. BBC News. 2020.

36 Ipsos MORI. Gender differences in attitudes towards Coronavirus. 2020. https://www.ipsos.com/sites/default/files/ct/news/documents/2020-05/fawcett_society_presentation.pdf (accessed 10 Jul 2020).

37 Ipsos MORI, The Fawcett Society. 6 in 10 women finding it harder to stay positive day-to-day due to Coronavirus: Women more likely to bearing the emotional and impact of the pandemic . Fawcett Soc. 2020.https://www.fawcettsociety.org.uk/Handlers/Download.ashx?IDMF=f173fde3-4edb-4c51-8453-7244e867ed90 (accessed 10 Jul 2020).

38 Thompson CW, Aspinall P, Bell S, *et al.* "It Gets You Away From Everyday Life": Local Woodlands and Community Use—What Makes a Difference? *Landsc Res* 2007;**30**:109–46. doi:10.1080/0142639042000324794

39 Burgess J. Focusing on Fear: The Use of Focus Groups in a Project for the Community Forest Unit, Countryside Commission. 1996.

40 O'Brien L, Morris J. Well-being for all? The social distribution of benefits gained from woodlands and forests in Britain. *Local Environ Int J Justice Sustain* 2014;**19**:356–83. doi:10.1080/13549839.2013.790354

41 Public Health England. Disparities in the risk and outcomes from COVID-19. London: 2020. www.facebook.com/PublicHealthEngland (accessed 26 Jul 2020).

42 Office for National Statistics. Deaths registered weekly in England and Wales, provisional: week ending 10 July 2020. ONS. 2020.https://www.ons.gov.uk/peoplepopulationandcommunity/birthsdeathsandmarriages/deaths/bulletins/deathsregisteredweeklyinenglandandwalesprovisional/latest#deaths-registered-by-age-group (accessed 26 Jul 2020).

43 TNS. Scotland's People and Nature Survey 2013/14. (Scottish Natural Heritage Commissioned Report No. 679). 2014.

44 Johnson B. Prime Minister's statement on coronavirus (COVID-19): 16 March 2020. GOV.UK. 2020.https://www.gov.uk/government/speeches/pm-statement-on-coronavirus-16-march-2020 (accessed 26 Jun 2020).

45 Office for National Statistics. Coronavirus and how people spent their time under lockdown. ONS. 2020.https://www.ons.gov.uk/economy/nationalaccounts/satelliteaccounts/bulletins/coronavirusandhowpeoplespenttheirtimeunderrestrictions/28marchto26april2020 (accessed 16 Jul 2020).

46 Honey-Rosés J, Anguelovski I, Chireh VK, *et al.* The impact of COVID-19 on public space: an early review of the emerging questions – design, perceptions and inequities. *Cities Heal* 2020;:1–17. doi:10.1080/23748834.2020.1780074

47 Ravenscroft N, Markwell S. Ethnicity and the integration and exclusion of young people through urban park and recreation provision. *Manag Leis* 2000;**5**:135–50. doi:10.1080/13606710050084838

48 Moore SA, Faulkner G, Rhodes RE, *et al.* Impact of the COVID-19 virus outbreak on movement and play behaviours of Canadian children and youth: a national survey. *Int J Behav Nutr Phys Act* 2020;**17**. doi:10.1186/s12966-020-00987-8

For peer review only - http://bmjopen.bmj.com/site/about/guidelines.xhtml

Figure legends:

Figure 1: Interaction between age and ethnicity for change in time spent visiting green space since movement restrictions were enforced.

For peer review only - http://bmjopen.bmj.com/site/about/guidelines.xhtml

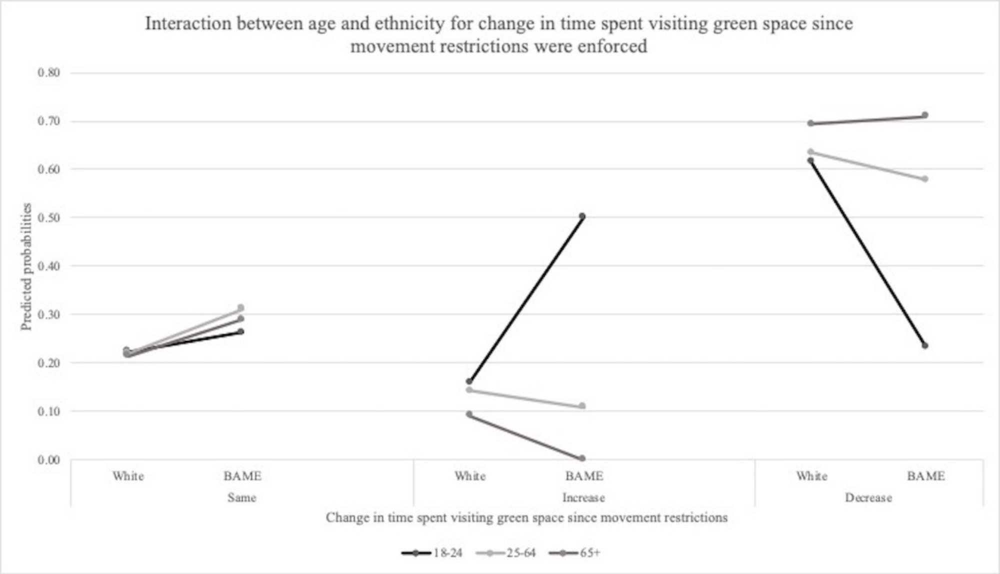

Figure 1: Interaction between age and ethnicity for change in time spent visiting green space since movement restrictions were enforced.

90x51mm (300 x 300 DPI)

Supplementary Materials

Supplementary Table 1: Category mergers of the response categories (ethnicity, age, social grade, sex and dog ownership).

	Original categories (Unweighted N)	New categories (Unweighted N)
**Ethnicity**	White British (1659)	White (1745)
	Any other white background (86)	
	White and Black Caribbean (6)	BAME (87)
	White and Black African (4)	
	White and Asian (8)	
	Any other mixed background (8)	
	Indian (17)	
	Pakistani (5)	
	Bangladeshi (4)	
	Any other Asian background (3)	
	Black Caribbean (4)	
	Black African (3)	
	Any other black background (3)	
	Chinese (10)	
	Other ethnic group (12)	
	Prefer not to say (19)	Missing (420)
	Not answered (401)	
**Age**	18-24 (165)	18-24 (165)
	25-34 (316)	25-64 (1497)
	35-44 (396)	
	45-54 (398)	
	55-64 (387)	
	65+ (590)	65+ (590)
**Social grade**	ABC1 (1419)	
	C2DE (833)	
**Sex**	Male (1018)	
	Female (1234)	
**Dog ownership**	Yes (616)	
	No (1636)	

For peer review only

Supplementary Table 2: Change in green space visit frequency since restrictions by individual demographic and socio-economic characteristics (unweighted).

	Visited green space in the year before restrictions		Visited green space after restrictions (only those who visited green space before restrictions)		Green space visitation change since lockdown		
	Yes (N=2045, 93.5%)	No (N=142, 6.4%)	Yes (N=1099, 53.8%)	No (N=943, 46.2%)	Decreased (N=1392, 63.3%)	Same (N=473, 21.5%)	Increased (N=333, 15.2%)
	% *(N)*	% *(N)*	% *(N)*	% *(N)*	% *(N)*	% *(N)*	% *(N)*
Sex							
**Male (ref)**	92.6% *(919)*	7.4% *(73)*	55.2% *(507)*	44.8% *(411)*	61.6% *(609)*	23.5% *(232)*	15% *(148)*
**Female**	94.2% *(1126)*	5.8% *(69)*	52.7% *(592)*	47.3% *(532)*	64.8% *(783)*	19.9% *(241)*	15.3% *(185)*
Social grade							
**ABC1 (ref)**	95.8% *(1328)*	4.2% *(58)*	56.7% *(753)*	43.3% *(574)*	62.9% *(871)*	18.8% *(260)*	18.3% *(254)*
**C2DE**	89.5% *(717)*	10.5% *(84)*	48.4% *(346)*	51.6% *(369)*	64.1% *(521)*	26.2% *(213)*	9.7% *(79)*
Age							
**18-24**	93.3% *(139)*	6.7% *(10)*	56.9% *(78)*	43.1% *(59)*	58.4% *(87)*	20.8% *(31)*	20.8% *(31)*
**25-64 (ref)**	93.6% *(1364)*	6.4% *(94)*	56.1% *(765)*	43.9% *(598)*	61.8% *(906)*	21.5% *(315)*	16.7% *(245)*
**65+**	93.4% *(542)*	6.6% *(38)*	47.2% *(256)*	52.8% *(286)*	68.4% *(399)*	21.8% *(127)*	9.8% *(57)*
Ethnicity							
**White (ref)**	93.6% *(1592)*	6.4% *(109)*	52.1% *(828)*	47.9% *(762)*	64.1% *(1098)*	22.5% *(385)*	13.4% *(229)*
**BAME**	84.1% *(69)*	15.9% *(13)*	47.8% *(33)*	52.2% *(36)*	57% *(45)*	30.4% *(24)*	12.7% *(10)*
Dog ownership							
**Don't own a dog/s (ref)**	93.1% *(1476)*	6.9% *(109)*	51.4% *(757)*	48.6% *(717)*	64.4% *(1027)*	19.5% *(311)*	16.1% *(256)*
**Own a dog/s**	94.5% *(569)*	5.5% *(33)*	60.2% *(342)*	39.8% *(226)*	60.4% *(365)*	26.8% *(162)*	12.7% *(77)*

Don't know/ can't recall responses were all excluded from analysis.

For peer review only

Supplementary Table 3: Change in green space experience since restrictions by individual demographic and socio-economic characteristics (unweighted).

	Using green spaces benefits my mental health more now			Miss social interaction in green spaces now			Do more physical activity in green spaces now		
	**Agree (N=721, 65.8%)**	**Neither (N=272, 24.8%)**	**Disagree (N=102, 9.3%)**	**Agree (N=590, 54.1%)**	**Neither (N=288, 26.4%)**	**Disagree (N=213, 19.5%)**	**Agree (N=322, 29.5%)**	**Neither (N=337, 30.9%)**	**Disagree(N=431, 39.5%)**
	% *(N)*		% *(N)*	% *(N)*		% *(N)*	% *(N)*		% *(N)*
Sex									
**Male (ref)**	59.6% *(300)*	30.8% *(155)*	9.5% *(48)*	45.8% *(231)*	30% *(151)*	24.2% *(122)*	27.4% *(137)*	35.4% *(177)*	37.2% *(186)*
**Female**	71.1% *(421)*	19.8% *(117)*	9.1% *(54)*	61.2% *(359)*	23.3% *(137)*	15.5% *(91)*	31.4% *(185)*	27.1% *(160)*	41.5% *(245)*
Social grade									
**ABC1 (ref)**	68% *(511)*	22.9% *(172)*	9.1% *(68)*	54.9% *(412)*	25.3% *(190)*	19.7% *(148)*	31.6% *(236)*	28.9% *(216)*	39.6% *(296)*
**C2DE**	61% *(210)*	29.1% *(100)*	9.9% *(34)*	52.2% *(178)*	28.7% *(98)*	19.1% *(65)*	25.1% *(86)*	35.4% *(121)*	39.5% *(135)*
Age									
**18-24**	67.5% *(52)*	19.5% *(15)*	13% *(10)*	64.9% *(50)*	22.1% *(17)*	13% *(10)*	48.7% *(38)*	16.7% *(13)*	34.6% *(27)*
**25-64 (ref)**	69% *(526)*	21% *(160)*	10% *(76)*	53.7% *(407)*	25.9% *(196)*	20.4% *(155)*	30.8% *(233)*	30.1% *(228)*	39.1% *(296)*
**65+**	55.9% *(143)*	37.9% *(97)*	6.2% *(16)*	52% *(133)*	29.3% *(75)*	18.8% *(48)*	20% *(51)*	37.6% *(96)*	42.4% *(108)*
Ethnicity									
**White (ref)**	62.8% *(518)*	27.3% *(225)*	9.9% *(82)*	51.6% *(423)*	28% *(230)*	20.4% *(167)*	26.5% *(218)*	32.3% *(266)*	41.2% *(339)*
**BAME**	75% *(24)*	21.9% *(7)*	3.1% *(1)*	51.5% *(17)*	30.3% *(10)*	18.2% *(6)*	38.7% *(12)*	38.7% *(12)*	22.6% *(7)*
Dog ownership									
**Don't own a dog/s (ref)**	68.2% *(514)*	23.9% *(180)*	8% *(60)*	52.5% *(395)*	27.1% *(204)*	20.5% *(154)*	34.5% *(259)*	28.7% *(215)*	36.8% *(276)*
**Own a dog/s**	60.7% *(207)*	27% *(92)*	12.3% *(42)*	57.7% *(195)*	24.9% *(84)*	17.5% *(59)*	18.5% *(63)*	35.9% *(122)*	45.6% *(155)*

Don't know/ can't recall responses were all excluded from analysis.

For peer review only

Supplementary Table 4: Significant interactions as predicted probabilities for visiting green space before restrictions on movement (ethnicity and social grade) and after the restrictions on movement were enforced (ethnicity and age).

	**Before restrictions on movement:**			**After restrictions on movement:**			
	**ABC1 (Higher social grade)**	**C2DE (Lower social grade)**	**p-value**	**18-24**	**25-64**	**65+**	**p-value**
**White**	0.96	0.89	**0.007**	0.42	0.53	0.48	**0.005**
**BAME**	0.82	0.88		0.82	0.49	0.32	

For peer review only

Supplementary Table 5: Significant interactions as predicted probabilities for change in green space visits following restrictions on movement (ethnicity and age) and the three experience outcomes (social grade and age).

Change in green space visits		18-24	25-64	65+	p-value
**White**	**Increase**	0.16	0.14	0.09	**0.015**
	**Neither**	0.22	0.22	0.22	
	**Decrease**	0.62	0.64	0.69	
**BAME**	**Increase**	0.50	0.11	0.00	
	**Neither**	0.26	0.31	0.29	
	**Decrease**	0.23	0.58	0.71	
**Mental Health**					
**ABC1**	**Agree**	0.79	0.70	0.56	**0.023**
	**Neither**	0.10	0.20	0.38	
	**Disagree**	0.11	0.10	0.06	
**C2DE**	**Agree**	0.26	0.64	0.53	
	**Neither**	0.33	0.27	0.41	
	**Disagree**	0.41	0.10	0.06	
**Social Interaction**					
**ABC1**	**Agree**	0.70	0.54	0.49	**0.008**
	**Neither**	0.08	0.27	0.28	
	**Disagree**	0.22	0.19	0.22	
**C2DE**	**Agree**	0.45	0.45	0.55	
	**Neither**	0.43	0.29	0.32	
	**Disagree**	0.13	0.26	0.13	
**Physical Activity**					

For peer review only

**ABC1**	**Agree**	0.41	0.31	0.18	**0.047**
	**Neither**	0.29	0.27	0.43	
	**Disagree**	0.30	0.42	0.39	
**C2DE**	**Agree**	0.45	0.25	0.16	
	**Neither**	0.18	0.39	0.33	
	**Disagree**	0.36	0.36	0.51	

For peer review only

STROBE Statement—Checklist of items that should be included in reports of ***cross-sectional studies***

	Item No	Recommendation	Page No
**Title and abstract**	1	(*a*) Indicate the study's design with a commonly used term in the title or the abstract	P1
		(*b*) Provide in the abstract an informative and balanced summary of what was done and what was found	P2
**Introduction**			
Background/rationale	2	Explain the scientific background and rationale for the investigation being reported	P3/4
Objectives	3	State specific objectives, including any prespecified hypotheses	P3/4
**Methods**			
Study design	4	Present key elements of study design early in the paper	P5/6
Setting	5	Describe the setting, locations, and relevant dates, including periods of recruitment, exposure, follow-up, and data collection	P5/6
Participants	6	(*a*) Give the eligibility criteria, and the sources and methods of selection of participants	P5/6
Variables	7	Clearly define all outcomes, exposures, predictors, potential confounders, and effect modifiers. Give diagnostic criteria, if applicable	P5-7
Data sources/ measurement	8*	For each variable of interest, give sources of data and details of methods of assessment (measurement). Describe comparability of assessment methods if there is more than one group	P4/5
Bias	9	Describe any efforts to address potential sources of bias	P4, 7
Study size	10	Explain how the study size was arrived at	P4/5
Quantitative variables	11	Explain how quantitative variables were handled in the analyses. If applicable, describe which groupings were chosen and why	P6-8
Statistical methods	12	(*a*) Describe all statistical methods, including those used to control for confounding	P7/8
		(*b*) Describe any methods used to examine subgroups and interactions	P8
		(*c*) Explain how missing data were addressed	P4-6
		(*d*) If applicable, describe analytical methods taking account of sampling strategy	P6-8
		(*e*) Describe any sensitivity analyses	-
**Results**			
Participants	13*	(a) Report numbers of individuals at each stage of study—eg numbers potentially eligible, examined for eligibility, confirmed eligible, included in the study, completing follow-up, and analysed	Supplementary Tables 1-3
		(b) Give reasons for non-participation at each stage	-
		(c) Consider use of a flow diagram	-

For peer review only - http://bmjopen.bmj.com/site/about/guidelines.xhtml

Descriptive data	14*	(a) Give characteristics of study participants (eg demographic, clinical, social) and information on exposures and potential confounders	P4-6, Supplementary Table 1
		(b) Indicate number of participants with missing data for each variable of interest	Table 1 + Supplementary Table 1
Outcome data	15*	Report numbers of outcome events or summary measures	Tables 2-4+ Supplementary Tables 2-5
Main results	16	(*a*) Give unadjusted estimates and, if applicable, confounder-adjusted estimates and their precision (eg, 95% confidence interval). Make clear which confounders were adjusted for and why they were included	P8-14
		(*b*) Report category boundaries when continuous variables were categorized	-
		(*c*) If relevant, consider translating estimates of relative risk into absolute risk for a meaningful time period	-
Other analyses	17	Report other analyses done—eg analyses of subgroups and interactions, and sensitivity analyses	P9/10
**Discussion**			
Key results	18	Summarise key results with reference to study objectives	P14/15
Limitations	19	Discuss limitations of the study, taking into account sources of potential bias or imprecision. Discuss both direction and magnitude of any potential bias	P17
Interpretation	20	Give a cautious overall interpretation of results considering objectives, limitations, multiplicity of analyses, results from similar studies, and other relevant evidence	P14-17
Generalisability	21	Discuss the generalisability (external validity) of the study results	P14, 17, 18
**Other information**			
Funding	22	Give the source of funding and the role of the funders for the present study and, if applicable, for the original study on which the present article is based	P18

*Give information separately for exposed and unexposed groups.

Note: An Explanation and Elaboration article discusses each checklist item and gives methodological background and published examples of transparent reporting. The STROBE checklist is best used in conjunction with this article (freely available on the Web sites of PLoS Medicine at http://www.plosmedicine.org/, Annals of Internal Medicine at http://www.annals.org/, and Epidemiology at http://www.epidem.com/). Information on the STROBE Initiative is available at www.strobe-statement.org.

For peer review only

For peer review only - http://bmjopen.bmj.com/site/about/guidelines.xhtml

BMJ Open

Change in time spent visiting and experiences of green space following restrictions on movement during the COVID-19 pandemic: A nationally representative cross-sectional study of UK adults.

Journal:	*BMJ Open*
Manuscript ID	bmjopen-2020-044067.R2
Article Type:	Original research
Date Submitted by the Author:	20-Jan-2021
Complete List of Authors:	Burnett, Hannah; University of Glasgow, MRC/CSO Social and Public Health Sciences Unit<br>Olsen, Jonathan; University of Glasgow, MRC/CSO Social and Public Health Sciences Unit<br>Nicholls, Natalie; University of Glasgow, MRC/CSO Social and Public Health Sciences Unit<br>Mitchell, Richard; University of Glasgow, MRC/CSO Social and Public Health Sciences Unit
**Primary Subject Heading**:	Public health
Secondary Subject Heading:	Mental health
Keywords:	MENTAL HEALTH, COVID-19, PUBLIC HEALTH

SCHOLARONE™
Manuscripts

BMJ

I, the Submitting Author has the right to grant and does grant on behalf of all authors of the Work (as defined in the below author licence), an exclusive licence and/or a non-exclusive licence for contributions from authors who are: i) UK Crown employees; ii) where BMJ has agreed a CC-BY licence shall apply, and/or iii) in accordance with the terms applicable for US Federal Government officers or employees acting as part of their official duties; on a worldwide, perpetual, irrevocable, royalty-free basis to BMJ Publishing Group Ltd ("BMJ") its licensees and where the relevant Journal is co-owned by BMJ to the co-owners of the Journal, to publish the Work in this journal and any other BMJ products and to exploit all rights, as set out in our licence.

The Submitting Author accepts and understands that any supply made under these terms is made by BMJ to the Submitting Author unless you are acting as an employee on behalf of your employer or a postgraduate student of an affiliated institution which is paying any applicable article publishing charge ("APC") for Open Access articles. Where the Submitting Author wishes to make the Work available on an Open Access basis (and intends to pay the relevant APC), the terms of reuse of such Open Access shall be governed by a Creative Commons licence – details of these licences and which Creative Commons licence will apply to this Work are set out in our licence referred to above.

Other than as permitted in any relevant BMJ Author's Self Archiving Policies, I confirm this Work has not been accepted for publication elsewhere, is not being considered for publication elsewhere and does not duplicate material already published. I confirm all authors consent to publication of this Work and authorise the granting of this licence.

For peer review only

Change in time spent visiting and experiences of green space following restrictions on movement during the COVID-19 pandemic: A nationally representative cross-sectional study of UK adults.

Hannah Burnett[1], Jonathan R Olsen[1], Natalie Nicholls[1], Richard Mitchell[1]
[1]MRC/CSO Social and Public Health Sciences Unit, University of Glasgow, Berkeley Square, 99 Berkeley Street, Glasgow, G3 7HR, United Kingdom.

Correspondence:
Hannah Burnett
MRC/CSO Social and Public Health Sciences Unit,
University of Glasgow,
Berkeley Square,
99 Berkeley Street,
Glasgow,
G3 7HR.
Email: h.burnett.1@research.gla.ac.uk

Key words: mental health; COVID-19; public health.

Word Count: 3,721
Tables: 4
Supplementary tables: 7

ABSTRACT

Objectives Green space positively influences health and well-being, however inequalities in use of green space are prevalent. Movement restrictions enforced due to the COVID-19 pandemic could have exacerbated existing inequalities regarding who visits green space. Therefore, this study aimed to explore how movement restrictions have changed the time spent visiting green space and experience of green space in the United Kingdom (UK) and how these differed by individual-level demographic characteristics.

Design and outcome measures A nationally representative cross-sectional survey administered through YouGov between 30th April–1st May 2020. Data were collected on the time spent visiting green space and change in the experience of green space, including missing social interaction, increased physical activity, and feeling greater mental health benefits in green space. Demographic information was collected on sex, age, ethnicity, social grade and dog ownership. Associations between specific outcome variables and predictors were assessed using logistic regression.

Setting United Kingdom, with population weights applied.

Participants 2,252 adults aged 18 years and over.

Results Overall, 63% of respondents reported a decrease in time spent visiting green space following movement restrictions. Lower social grade respondents were less likely to visit green space before *and* after restrictions were enforced (OR:0.35 (95% CI:0.24-0.51); OR:0.77 (95% CI:0.63-0.95)). Female respondents were more likely than male respondents to agree that green space benefitted their mental health more following restrictions (PP:0.70 vs. 0.59). Older (65+) respondents were less likely than middle age (25-64 years) respondents to have visited green space following the restrictions (OR:0.79 (95% CI:0.63-0.98)).

Conclusions Inequalities in green space use were sustained, and possibly exacerbated, during movement restrictions. Our findings emphasise the importance of green spaces remaining open globally in any future 'lockdowns'/pandemics. Further investigation is required to determine how visit patterns and experiences change through the different stages of the COVID-19 pandemic in the UK.

(Word Count: 300)

Strengths and Limitations of this study

- Our data are currently the only existing data covering change in the time spent visiting green space and experiences of green space for the UK population following the movement restrictions enforced due to the COVID-19 pandemic.
- The sample is nationally representative of UK adults.
- Collecting data on *both* time spent visiting green space and change in experience of green space during the movement restrictions is a strength of this study, compared to other surveys exploring change in green space use during the COVID-19 pandemic, including the Google COVID-19 Community Mobility Reports and Natural England's People and Nature Survey.

- The data collected for this study were from a cross-sectional survey, therefore it is not possible to demonstrate causality between change in green space use and experiences and the demographic data.
- Certain variable response categories were necessarily recategorised for analysis, which meant that some distinctiveness across groups were lost, particularly for the ethnicity and age variables.

INTRODUCTION

Evidence suggests that exposure to green space has a positive influence on health and well-being.[1,2] Green space use is associated with increased levels of social interaction and physical activity, and decreased levels of all-cause mortality, loneliness and stress.[3–5] Additionally, there is evidence that contact with green space may disproportionately benefit disadvantaged populations, reducing health inequalities and therefore weakening the effects of poverty – known as the 'equigenic' effect.[6,7]

The health impacts of contact with green spaces are quite well studied by both observational and experimental designs. There is rather less literature on the levels of, motivations for, and barriers to visiting green spaces in the first place. Recent surveys by Natural England and Scottish Natural Heritage have found that 74% of the English population and 70% of the Scottish population visit green space 'frequently' (once a month or more often). In both countries, the top three reasons stated for frequent green space use were health/exercise, dog walking, and to relax/unwind.[8,9] Further research exploring the motivations and reasons for visiting green space found that visitation frequency is affected by an individual's feelings of nature connectedness, as well as their childhood exposure to nature.[10–12]. It is important to note that substantial inequalities in green space use have also been reported, with studies finding that females, older people and those from less advantaged socioeconomic positions (SEP) are more likely to be infrequent users than their male, younger and higher SEP counterparts.[9,13–15]

In 2020, the United Kingdom (UK) experienced major disruption to everyday life due to the novel coronavirus disease 2019 (COVID-19) pandemic. COVID-19 is an infectious disease first identified in the city of Wuhan, China in December 2019.[16] As a response to the COVID-19 outbreak, the UK announced a series of movement restrictions from 23rd March 2020. These included rules designed to increase social distancing, meaning that people could only leave their households to make 'essential trips' for food, medication and exercise.[17] Recent studies have highlighted the negative effects of COVID-19 on the UK population's mental health and wellbeing, which are likely to be profound and long-lasting.[18] Research exploring the wider health effects of COVID-19 suggest that the negative indirect effects are being borne disproportionately by people who have fewer resources and poorer health.[19] If natural environments usually act to mitigate the connections between adversity and poor health,[6,7] it is important to assess the extent to which lockdown affected both use and experience of such environments. Future lockdowns and movement restrictions are highly likely as second, third and perhaps fourth waves of the pandemic take place around the world.

The aim of this research was to explore changes in the time spent visiting green space and experience of visits to green space among the UK population following movement restrictions. The sociodemographic characteristics focused on were those identified by the literature as being most consistent markers of inequality in use. The research questions were:

For peer review only - http://bmjopen.bmj.com/site/about/guidelines.xhtml

1) How did time spent visiting green space change following movement restrictions, compared with before, and for whom?
2) How did experiences of visits to green space change following the restrictions, and did any change vary by sex, social grade, age, ethnicity and dog ownership? Specifically,
   a) Did respondents feel that green space benefitted their mental health more since restrictions than before?
   b) Did respondents miss social interaction in green space more following movement restrictions?
   c) Had physical activity increased following movement restrictions?

METHODS

Survey design and sample

An online cross-sectional survey was administered by YouGov between 30th April and 1st May 2020.[20] Questions were answered by a sample of 2,252 adults from the UK (aged 18 and over). The sample was drawn from a panel of over 800,000 individuals who specifically opted in to participate in online research activities. Sample members were randomly selected from the panel and sent an email providing a survey link. Table 1 shows the themes and specific survey instruments analysed in this paper. Only the respondents that visited green space following movement restrictions were asked the questions regarding change in experience of green space. Demographic information about participants was also collected (including sex, age and social grade, which was classified by occupation).[21,22] Although the sample was reasonably large, small numbers in variable response categories necessitated some category mergers (Table 1 and Supplementary Table 1).

The survey covered adults from across the UK, with respondents from England (N=1,875), Scotland (N=209), Wales (N=107), and Northern Ireland (N=61). Weightings were applied to render the sample representative of UK adults (detailed below). When the survey was distributed, the same movement restrictions were implemented across the UK. These included only leaving home for limited purposes, such as medical needs, shopping for basic necessities (food and medicine), and exercising once a day alone/with members of your household.[23] Since then, the individual parliaments/assemblies representing these countries have imposed different COVID-19 policies.

Table 1: Survey themes and specific items analysed, including variables recategorised.

Themes	Question/statement	Potential responses	Recategorised
Change in the amount of time spent visiting green space	*"Did you EVER visit a green space in the year before the movement restrictions were enforced in the UK?"*	Yes, I did<br>No, I didn't<br>Don't know/ can't recall	-<br><br>*Don't know/can't recall excluded (N=80, 3.5%)*
	"Please think about your behaviour since the UK enforced a 'lock down' to restrict movement, as a result of the current Coronavirus (COVID-19) outbreak (i.e. since 23rd March 2020). Have you visited a green space since the movement	Yes, I have<br>No, I haven't<br>Don't know/ can't recall	-

	restrictions have been enforced in the UK?"		*Don't know/can't recall excluded (N=43, 1.9%)*
	"How much, if at all, has the amount of time that you have spent visiting green spaces changed since the 'lock down' movement restrictions began (i.e. 23rd March 2020) compared to before?"	Increased a lot<br>Increased a little<br>No difference<br>Decreased a little<br>Decreased a lot<br>Don't know	'Increased' (increased a lot and increased a little)<br>'Same' (no difference)<br>'Decreased' (decreased a lot and decreased a little)<br><br>*Don't know excluded (N=74, 3.3%)*
Experience change (If respondent had visited a green space since the movement restrictions were enforced)	*"I feel that being in green spaces benefits my mental health more now, than before the movement restrictions were in place"*	Strongly agree<br>Slightly agree<br>Neither agree nor disagree<br>Slightly disagree<br>Strongly disagree<br>Don't know/ can't recall	'Agree' (strongly agree and slightly agree)<br>'Neither' (neither agree nor disagree)<br>'Disagree' (slightly disagree and strongly disagree)<br><br>*Don't know/can't recall excluded (N=7, 0.6%)*
	"I have missed seeing and/ or talking with people in green spaces since the movement restrictions were introduced"	Strongly agree<br>Slightly agree<br>Neither agree nor disagree<br>Slightly disagree<br>Strongly disagree<br>Don't know/ can't recall	'Agree' (strongly agree and slightly agree)<br>'Neither' (Neither agree nor disagree)<br>'Disagree' (slightly disagree and strongly disagree)<br><br>*Don't know/can't recall excluded (N=8, 0.8%)*
	"I do more physical activity in green spaces now, than I did before the movement restrictions were introduced"	Strongly agree<br>Slightly agree<br>Neither agree nor disagree<br>Slightly disagree<br>Strongly disagree<br>Don't know/ can't recall	'Agree' (strongly agree and slightly agree)<br>'Neither' (Neither agree nor disagree)<br>'Disagree' (slightly disagree and strongly disagree)<br><br>*Don't know/can't recall excluded (N=9, 0.8%)*

Don't know/ can't recall responses were all excluded from analysis (weighted counts and proportions reported above).

Demographic variables

Individual level demographic and socio-economic characteristics were captured from the survey, as follows: sex (male, female); age group (18-24 years, 25-64 years, 65+ years); ethnicity (White, Black, Asian and minority ethnic (BAME)); dog ownership (Yes, No); and social grade (ABC1, C2DE), derived by YouGov from combined occupational social grade categories. ABC1 was the higher social grade, and included non-manual workers, for example, senior managers and owners of small establishments. C2DE was the lower social grade, and included all manual workers, for example, shop assistants and labourers.[24] Hereafter, social grade will be described as higher and lower social grade.

Patient and Public Involvement

There was no direct patient or public involvement in this study.

For peer review only - http://bmjopen.bmj.com/site/about/guidelines.xhtml

Analyses

Descriptive statistics

The count and proportion of respondents who had: visited green space before and after movement restrictions were enforced; increased or decreased visitation; and agreed or disagreed with the three change in experience statements (Table 1) are presented. These were also explored by sex, age, ethnicity, dog ownership and social grade.

Statistical analysis

Binary logistic regression analyses were conducted to assess the association between the individual predictor variables and the following two outcomes:

1. having visited green space in the year before movement restrictions were enforced;
2. having visited green space since movement restrictions were enforced, with this analysis being restricted to those who had reported visiting green space before movement restrictions.

The associations between the individual predictor variables and each of the following outcomes were also assessed using multinomial logistic regression or, if appropriate, ordinal logistic regression, with results presented as predicted probabilities (PP):

1. Change in green space visitation following movement restrictions;
2. Levels of agreement that green space benefits their mental health more now (since movement restrictions compared to before);
3. Levels of agreement that they miss seeing/talking to people in green space now (since movement restrictions compared to before); and
4. Levels of agreement that they do more physical activity in green space now (since movement restrictions compared to before).

The results of the binary logistic regression analyses were expressed as odds ratios (OR) with 95% confidence intervals (CIs). Other results are presented as predicted outcome group probabilities for each variable. Predicted probabilities (PP) can be interpreted as a an indicator of likelihood, so that the closer the value is to 1.0, the greater the likelihood. We opted to present these results as predicted probabilities as they better illustrate not only the size of the association between the predictor variable and response category, but also the difference in this *between* variables. For example, the likelihood of men either decreasing, maintaining or increasing their time in green space can be more easily compared to the likelihood of those in the higher social grade category decreasing, maintaining or increasing their time in green space using predicted probability than with an OR or risk ratio (RR). This is because an OR or RR is expressed relative to the reference category. For those who prefer a RR, these are provided in the supplementary material (Supplementary Tables 2-5).

Univariate models for each predictor were conducted first, followed by a fully adjusted model containing all predictors for each outcome. Weightings were calculated by YouGov, with the final data weighted to match the national profile of all adults aged 18 and over and applied during analyses to render the sample representative of UK adults.[22] All analyses were conducted in R v3.5.1,[25], and the *brant* package,[26], was used to check that the

proportionality of odds assumption for ordinal logistic regression was not violated.[27] A full R script is available on GitHub.[28]

RESULTS

Descriptive statistics

93% of all respondents had visited green space in the year before movement restrictions were enforced. In contrast, 53% of respondents reported visiting green space following movement restrictions. 63% of respondents reported that the amount of time they spent in green space had decreased since movement restrictions compared to before, with 15% reporting an increase, and 22% reporting no difference in the time spent visiting green space (Supplementary Table 6).

A greater proportion of respondents agreed (65%) than disagreed (10%) or neither agreed nor disagreed (25%) that green space benefited their mental health more following movement restrictions compared to before. More respondents agreed (54%) that they missed social interaction in green space more since movement restrictions, than disagreed (19%) or neither agreed nor disagreed (27%). 39% of respondents disagreed that they had increased physical activity in green space since movement restrictions, compared to 29% who agreed and 32% that neither agreed nor disagreed (Supplementary Table 7).

Change in visitation time

Visiting green space before movement restrictions

In the adjusted logistic regression model for visiting green space in the year before movement restrictions were enforced (Table 2), only two variables had significant associations ($p<0.05$). Lower social grade respondents (OR:0.35 (95%CI:0.24-0.51)) and BAME (OR:0.43 (95%CI:0.23-0.80)) respondents had lower odds of visiting green space before movement restrictions, compared to higher social grade and white respondents.

Visiting green space following movement restrictions

Respondents in the lower social grade group were less likely to have visited green space than respondents in the higher social grade group (OR:0.77 (95%CI:0.63-0.95)) (Table 2). Older respondents (65+) were also less likely than middle age respondents (25-64) to have visited green space following the movement restrictions (OR:0.79 (95% CI:0.63-0.98)). Respondents who owned a dog/s were more likely than respondents who did not to have visited green space after movement restrictions were enforced (OR:1.42 (95%CI:1.14-1.78)).

Change in time spent visiting green space

Following lockdown, changes in the time spent visiting green space was found to differ by demographic group. The likelihood of spending more time in greenspace was found to differ by social grade. Lower social grade respondents were less likely to report spending more time in green space following movement restrictions than higher social grade respondents (PP:0.09 vs. 0.16) (Table 3). Age was also found to be associated with change in time spent in green space. Older respondents (aged 65+) were the least likely to report increased green

space visits (PP:0.09(65+) compared to younger groups (PP: 0.14(25-64) and 0.21(18-24)). Females were more likely to report decreased green space visits compared to males (PP:0.67 vs. 0.62), as well as being less likely to report no change in visit time (PP:0.20 vs. 0.25)(Table 3). Finally, respondents without a dog/s were slightly more likely to report decreased green space visitations compared to dog owners(PP:0.66 vs. 0.62), and less likely to sustain their frequency of visitation (PP:0.21 vs. 0.28).

For peer review only - http://bmjopen.bmj.com/site/about/guidelines.xhtml

Table 2: Adjusted binary logistic regression models predicting green space visit before and after the movement restrictions were enforced. OR = odds ratio; CI = confidence intervals.

	**Visited green space in the year before restrictions (yes)**		**Visited green space after restrictions (yes & visited green space before restrictions)**	
	Adjusted		Adjusted	
	OR (95% CI)	p-value	OR (95% CI)	p-value
Sex				
**Male (ref)**				
**Female**	1.35 (0.94-1.95)	0.109	0.88 (0.72-1.07)	0.205
Social grade				
**ABC1 (ref)**				
**C2DE**	0.35 (0.24-0.51)	*<0.001*	0.77 (0.63-0.95)	*0.013*
Age				
**18-24**	2.92 (1.00-8.57)	0.051	0.81 (0.54-1.21)	0.297
**25-64 (ref)**				
**65+**	1.22 (0.80-1.85)	0.353	0.79 (0.63-0.98)	*0.035*
Ethnicity				
**White (ref)**				
**BAME**	0.43 (0.23-0.80)	*0.007*	1.06 (0.67-1.68)	0.799
Dog ownership				
**No (ref)**				
**Yes**	1.29 (0.83-2.00)	0.260	1.42 (1.14-1.78)	*0.002*

For peer review only - http://bmjopen.bmj.com/site/about/guidelines.xhtml

Table 3: Multinomial logistic regression models: Predicted probabilities (likelihoods) of being in each outcome group for change in time visiting green space and levels of agreement that green space benefits their mental health more now (since movement restrictions were enforced compared to before). The p-values presented reflect the significance of each factor in the model.

		**Change in time spent visiting green space**				**Using green spaces benefits my mental health more now**			
		**Decreased**	**Same**	**Increased**	**p-value**	**Agree**	**Neither**	**Disagree**	**p-value**
Sex	**Male**	0.62	0.25	0.13	*0.041*	0.59	0.31	0.10	*0.004*
	**Female**	0.67	0.20	0.12		0.70	0.22	0.09	
Social grade	**ABC1**	0.65	0.19	0.16	*<0.001*	0.68	0.24	0.09	*0.048*
	**C2DE**	0.64	0.27	0.09		0.59	0.31	0.10	
Age	**18-24**	0.57	0.22	0.21	*0.004*	0.55	0.20	0.25	*<0.001*
	**25-64**	0.63	0.22	0.14		0.68	0.22	0.10	
	**65+**	0.69	0.22	0.09		0.55	0.38	0.06	
Ethnicity	**White**	0.22	0.65	0.12	0.167	0.64	0.26	0.10	0.063
	**BAME**	0.30	0.56	0.14		0.73	0.25	0.02	
Dog ownership	**No**	0.66	0.21	0.13	*0.003*	0.67	0.24	0.09	0.117
	**Yes**	0.62	0.28	0.10		0.59	0.30	0.11	

For peer review only - http://bmjopen.bmj.com/site/about/guidelines.xhtml

Table 4: Multinomial logistic regression models: Predicted probabilities (likelihoods) of each levels of agreement that respondents missed seeing/talking to people in green space more since movement restrictions were enforced compared to before, and levels of agreement that respondents do more physical activity in green space following the movement restrictions. The p-values presented reflect the significance of each factor in the model.

		**Miss social interaction in green spaces now**				**Do more physical activity in green spaces now**			
		**Agree**	**Neither**	**Disagree**	**p-value**	**Agree**	**Neither**	**Disagree**	**p-value**
Sex	**Male**	0.45	0.30	0.25	*<0.001*	0.24	0.37	0.40	0.096
	**Female**	0.58	0.26	0.16		0.27	0.29	0.43	
Social grade	**ABC1**	0.53	0.27	0.20	0.256	0.27	0.31	0.42	0.307
	**C2DE**	0.48	0.31	0.21		0.23	0.36	0.41	
Age	**18-24**	0.59	0.23	0.18	0.672	0.44	0.23	0.33	*0.002*
	**25-64**	0.51	0.28	0.21		0.29	0.31	0.40	
	**65+**	0.53	0.30	0.18		0.18	0.38	0.45	
Ethnicity	**White**	0.52	0.28	0.20	0.802	0.25	0.32	0.42	0.063
	**BAME**	0.52	0.31	0.16		0.33	0.44	0.22	
Dog Ownership	**No**	0.50	0.30	0.20	0.295	0.31	0.30	0.39	*<0.001*
	**Yes**	0.55	0.25	0.20		0.17	0.38	0.46	

For peer review only - http://bmjopen.bmj.com/site/about/guidelines.xhtml

Change in visit experience

Mental health benefits

Females were more likely to agree than males that being in green space benefitted their mental health more following movement restrictions than before (PP:0.70 vs. 0.59). Higher social grade respondents were more likely to agree than lower social grade respondents (PP:0.68 vs. 0.59). Younger respondents were more likely to disagree that being in green space benefitted their mental health more following movement restrictions than before, whilst older respondents were less likely to disagree (PP:0.25(18-24) vs. 0.06(65+) and 0.10(25-64))(Table 3).

Missed social interaction

Female respondents were more likely to agree that they missed seeing and talking with other people in green space since movement restrictions compared to before than male respondents (PP:0.58 vs. 0.45)(Table 4).

Increased physical activity

Older respondents were less likely to agree that they had increased physical activity following movement restrictions (PP:0.18(65+) vs. 0.20(25-64) and 0.44(18-24)). Respondents who owned a dog/s were less likely than respondents without a dog/s to agree (PP:0.17 vs. 0.31)(Table 4).

DISCUSSION

Our findings suggest that inequalities in use of green space between demographic groups were sustained following movement restrictions, with lower social grade individuals less likely than higher social grade individuals to have visited green space before and since movement restrictions. Other existing inequalities in use were possibly exacerbated in the month after movement restrictions were enforced, with females being more likely to report a decrease in visits following movement restrictions.

The proportion of respondents who visited green space before and following movement restrictions decreased, from 93% to 53%. This was consistent with Natural England's findings from April 2020 where 49% of English adults reported green space visits in the previous two weeks.[29] Natural England conducted an online panel survey in April 2020 (N=2,083), the survey covered only the English population, collecting information on the frequency of time in green and natural spaces in the 12 months, and then 2 weeks, prior to the survey.[30] The survey did not directly ask respondents about whether they felt their experiences within green space had changed following movement restrictions. A similar study focused on change in time spent visiting parks using the Google COVID-19 Community Mobility Reports covering 620 counties across the United States(USA). They found a lower percentage decrease in park visits compared to our findings, reporting a 17-35% decrease in visits between 15th March-9th May 2020.[31] This difference may be explained by the focus on parks alone rather than different types of green space. However, additional research exploring the Google COVID-19 Community Mobility Reports found that

For peer review only - http://bmjopen.bmj.com/site/about/guidelines.xhtml

from 16th February-29th March 2020 park use decreased by 90% in Catalonia, 7% in Oslo, and 79% in New York County. In Stockholm, park use increased by 24% in the same timeframe.[32] This could be explained by Sweden having a less stringent approach to movement restrictions and instead relying on ‘self-responsibility’ to prevent the population having to restrict movement and stay at home.[33] The Swedish population may also be more culturally attuned to seeking time in nature to combat stress.

Females and older individuals were more likely to have reported decreased visits to green space following movement restrictions. These findings corroborate with existing data collected before movement restrictions.[9,13,14] Boyd *et al.* analysed the Natural England ‘Monitor of Engagement with the Natural Environment’ survey (N=63,890) with a focus on infrequent use. They found that females and older adults in England were more likely to be infrequent visitors.[9] Cohen *et al.* explored physical activity levels in Los Angeles’ parks (N=1,318). They reported that age and sex were predictors of park use, with <5% of park users over 60 years old, and males using parks more often than females (62% vs. 38%).[13]

Our findings suggested that existing patterns of gender inequality in use were sustained and potentially exacerbated due to fears arising from the COVID-19 pandemic. For example, an Ipsos MORI survey was conducted in April 2020, collecting data on gender differences in British attitudes towards COVID-19. They found that women were more likely to report following government rules to avoid leaving their home (78%) than men (68%) and felt more uncomfortable about returning to “normal”(N=1,000).[34,35]

Previous studies have found that females feel more vulnerable than males in green spaces, especially without company.[14,15,36–39] There are few quantitative studies focusing on sex and the importance of social interaction as a motivation behind green space use. We found that females were more likely to agree that they missed seeing/talking with others in green space than males. This could be explained by the nature of the movement restrictions at this time, particularly the inability to socially interact with individuals outside your household in green space.

Given older age is probably the most important risk factor for an adverse outcome from COVID-19,[40,41] it was not surprising that older individuals were less likely to report an increase in green space visits and to agree that they were doing more physical activity following movement restrictions. These findings corroborate with the majority of research exploring older age and green space use before the movement restrictions.[9,13,42] Older people in the UK have been found to be at particular risk of social isolation during social distancing, being less likely to use online communications and more likely to live alone than younger individuals.[19] Our findings also show that older people are less likely to have used green space during lockdown, further emphasising this point. The significant decrease in green space visits following movement restrictions for older respondents could be explained by the government advice for over 70s and those with an underlying health condition to shield, minimise interaction and stay at home, for around 12 weeks from 21st March 2020.[43]

Lower social grade respondents reported little or no change in visitation to green space, with time spent visiting green space remaining low both before and after movement restrictions. This is supported by previous studies exploring the association between socio-economic position (SEP) and green space use.[9,15,42] One possible explanation is a lack of interest in visiting green space reported by lower SEP individuals.[9] Additionally, the social grade

For peer review only - http://bmjopen.bmj.com/site/about/guidelines.xhtml

variable was categorised by occupation, and it was likely that individuals categorised as lower social grade were working in manual or service occupations.[24] They may have continued at their usual workplace/working hours during the COVID-19 pandemic. Findings from the Office for National Statistics support this explanation, with 30.5% of employees in the bottom three income deciles (monthly earnings of up to £1,450) considered as key workers in March/April 2020, compared to 26.4% in the top three income deciles (monthly earnings of up to £3,250).[44] Similarly, existing international research on public space use during COVID-19 restrictions worldwide state that skilled workers in the knowledge economy have shifted easily into online work from home, and can therefore make more use of green spaces during the movement restrictions.[45]

The results for dog owners suggest that the movement restrictions have had an overall negative impact on their experiences within green space. Although dog owners were more likely to have visited green space following the movement restrictions than those that did not own a dog, they were less likely to agree that they had increased physical activity and more likely to have decreased visitations following the restrictions. These results differ from research undertaken in Canada that reported findings that dog ownership was associated with more outdoor play and less indoor play in Canadian youth at the start of the pandemic.[46] However, the focus on youth rather than adults may explain this difference in findings. Instead, the difference found in visits after restrictions were enforced may be due to dog owners having to walk their dog/s in green spaces *despite* the pandemic restrictions. In comparison between our results and those of other studies, we are mindful of the difference between number of visits and time spent in green spaces as measures of ‘use’. It would be entirely possible to increase one at the expense of the other and our data were not well suited to unpacking the relationship between them.

Strengths and limitations

Our study has several strengths; to our knowledge, currently this is the only data covering UK population change in use and experiences within green space at the start of the movement restrictions from 23rd March 2020. This means that the data could provide timely information to local and national governments across the UK. It is important to understand the effects of the initial movement restrictions to generate policy recommendations for any future movement restrictions. The rapid collection of data, just over a month after the movement restrictions were introduced, reduces risk of recall bias. The sample is nationally representative of UK adults, with weightings calculated by YouGov and applied to all analyses, reducing risk of selection bias.[22]

A further strength of our study is providing quantitative data on the importance of social interaction as a driver for using green space, which is under-reported in the literature. We analysed social grade at an individual level, which provides greater detail and accuracy of the respondent’s SEP than at neighbourhood level.

There are some caveats. The survey design was cross-sectional, and we are unable to demonstrate causality between change in green space use and experiences and the demographic data. Additionally, the data on use and experiences are self-reported and therefore subjective. Certain variable response categories had to be recategorised in order to analyse them. This is particularly evident in the ethnicity and age variables, where some distinctiveness across groups was lost. The variable best capturing change in behaviour

measured time spent in green space, but this made it difficult to understand whether respondents traded off time and numbers of visits.

We encourage future research to explore the reasons *why* many patterns and changes in use of green space since movement restrictions have emerged. It would also be interesting to explore the change in patterns of use before and following movement restrictions for those that did not visit before the movement restrictions but did visit green space following restrictions.

CONCLUSION

Our study provides novel evidence to suggest that green space use and experiences were profoundly affected during the first month of movement restrictions in the UK which were part of the response to COVID-19. Our findings suggested that inequalities in use were sustained, with lower social grade individuals less likely to visit green space than higher social grade individuals before and following movement restrictions. It is possible that these inequalities were exacerbated, as females and older individuals were the groups most likely to have decreased visits following movement restrictions. Although these findings reflect the UK population's experience during the movement restrictions, they could be compared with countries such as Norway, USA, and Spain where green space visits also decreased. We believe that these findings emphasise the need for green spaces to remain open in any future 'lockdowns' and for governments to actively encourage individuals to utilise these spaces to support their mental and physical health during subsequent waves of the pandemic. Further investigation is required to support these findings and how they may change through the stages of the COVID-19 pandemic and the relaxing, and potential re-introduction, of movement restrictions.

COMPETING INTERESTS
None declared.

DATA SHARING STATEMENT
We have made our research dataset publicly available (Datacite DOI: 10.5525/gla.researchdata.1038 - embargoed until 31/10/2023).

CONTRIBUTORS
HB, JRO and RM designed the study and survey; HB conducted data analysis which was reviewed by NN; HB wrote the first draft of the paper; all authors contributed to interpreting the results and revising the draft. The corresponding author attests that all listed authors meet authorship criteria and that no others meeting the criteria have been omitted.

FUNDING STATEMENT
The authors (HB, JRO, NN & RM) are part of the Places and Health Programme at the MRC/CSO Social and Public Health Sciences Unit (SPHSU), University of Glasgow, supported by the Medical Research Council (MC_UU_12017/10) and the Chief Scientist Office (SPHSU10).

References:

1 Barton J, Pretty J. What is the Best Dose of Nature and Green Exercise for Improving Mental Health? A Multi-Study Analysis. *Environ Sci Technol* 2010;**44**:3947–55.

For peer review only - http://bmjopen.bmj.com/site/about/guidelines.xhtml

doi:10.1021/es903183r

2 Douglas O, Lennon M, Scott M. Green space benefits for health and well-being: A life-course approach for urban planning, design and management. *CITIES* 2017;**66**:53–62. doi:10.1016/j.cities.2017.03.011

3 Twohig-Bennett C, Jones A. The health benefits of the great outdoors: A systematic review and meta-analysis of greenspace exposure and health outcomes. *Environ Res* 2018;**166**:628–37. doi:10.1016/j.envres.2018.06.030

4 Maas J, Van Dillen SME, Verheij RA, *et al.* Social contacts as a possible mechanism behind the relation between green space and health. *Health Place* 2009;**15**:586–95. doi:10.1016/j.healthplace.2008.09.006

5 Wells N, Evans G. NEARBY NATURE A Buffer of Life Stress Among Rural Children. *Environ Behav* 2003;**35**:311–30. doi:10.1177/0013916503251445

6 Frumkin H, Bratman GN, Breslow SJ, *et al.* Nature Contact and Human Health: A Research Agenda. *Environ Health Perspect* 2017;**125**. doi:10.1289/EHP1663

7 Mitchell RJ, Richardson EA, Shortt NK, *et al.* Neighborhood Environments and Socioeconomic Inequalities in Mental Well-Being. *Am J Prev Med* 2015;**49**:80–4. doi:10.1016/j.amepre.2015.01.017

8 Wilson V, Seddon B. Scotland’s People and Nature Survey 2017/18 – outdoor recreation and health modules. (Scottish Natural Heritage Research Report No. 1062). Inverness: 2018.

9 Boyd F, White MP, Bell SL, *et al.* Who doesn’t visit natural environments for recreation and why: A population representative analysis of spatial, individual and temporal factors among adults in England. *Landsc Urban Plan* 2018;**175**:102–13. doi:10.1016/j.landurbplan.2018.03.016

10 Bell SL, Phoenix C, Lovell R, *et al.* Green space, health and wellbeing: making space for individual agency. *Health Place* 2014;**30**:287–92. doi:10.1016/j.healthplace.2014.10.005

11 Lin BB, Fuller RA, Bush R, *et al.* Opportunity or Orientation ? Who Uses Urban Parks and Why. 2014;**9**:1–7. doi:10.1371/journal.pone.0087422

12 Ward Thompson C, Aspinall P, Montarzino A. The Childhood Factor Adult Visits to Green Places and the Significance of Childhood Experience. *Environ Behav* 2008;**40**:111–43. doi:10.1177/0013916507300119

13 Cohen DA, McKenzie TL, Sehgal A, *et al.* Contribution of Public Parks to Physical Activity. *Am J Public Health* 2007;**97**:509–14. doi:10.2105/AJPH.2005.072447

14 Lee ACK, Maheswaran R. The health benefits of urban green spaces: A review of the evidence. *J Public Health (Bangkok)* 2011;**33**:212–22. doi:10.1093/pubmed/fdq068

15 Morris J, O’Brien E, Ambrose-Oji B, *et al.* Access for all? Barriers to accessing woodlands and forests in Britain. *Local Environ Int J Justice Sustain* 2011;**16**:375–96. doi:10.1080/13549839.2011.576662

16 Wynants L, Van Calster B, Bonten MMJ, *et al.* Prediction models for diagnosis and prognosis of covid-19 infection: systematic review and critical appraisal . *BMJ* 2020;**369**. doi:10.1136/bmj.m1328

17 Johnson B. Prime Minister’s statement on coronavirus (COVID-19): 23 March 2020. GOV.UK. 2020.https://www.gov.uk/government/speeches/pm-address-to-the-nation-on-coronavirus-23-march-2020 (accessed 7 Jul 2020).

18 O’Connor RC, Wetherall K, Cleare S, *et al.* Mental health and wellbeing during the COVID-19 pandemic: longitudinal analyses of adults in the UK COVID-19 Mental Health & Wellbeing study. *Br J Psychiatry* 2020;:1–17. doi:10.1192/bjp.2020.212

19 Douglas M, Katikireddi SV, Taulbut M, *et al.* Mitigating the wider health effects of covid-19 pandemic response. *BMJ* 2020;**369**. doi:10.1136/bmj.m1557

For peer review only - http://bmjopen.bmj.com/site/about/guidelines.xhtml

20 YouGov. About YouGov Company. https://yougov.co.uk/about/ (accessed 8 Jun 2020).

21 YouGov and ESOMAR (World Association for Social Opinion and Market Research). ESOMAR 28: 28 Questions to help Online Research Buyers. The Netherlands: 2012. http://cdn.yougov.com/cumulus_uploads/document/t3r5k565j5/ESOMAR_28.pdf (accessed 8 Jun 2020).

22 YouGov. Panel Methodology. https://yougov.co.uk/about/panel-methodology/ (accessed 8 Jun 2020).

23 Cabinet Office. Staying at home and away from others (social distancing). London: 2020. https://www.gov.uk/government/publications/full-guidance-on-staying-at-home-and-away-from-others (accessed 8 Oct 2020).

24 The Market Research Society (MRS). Definitions employed in Social Grading. MRS. https://www.mrs.org.uk/pdf/Definitions used in Social Grading based on OG7.pdf (accessed 2 Jul 2020).

25 R Core Team. R: A Language and Environment for Statistical Computing. 2018.https://www.r-project.org/

26 Schlegel BE. Package brant. https://benjaminschlegel.ch/en/r/brant/ (accessed 29 Oct 2020).

27 Brant R. Assessing proportionality in the proportional odds model for ordinal logistic regression. *Biometrics* 1990;**46**:1171–8.

28 Burnett H. Change in green space use and experiences following UK restrictions on movement. GitHub. 2020.https://github.com/hannahburnett/greenspace-covid_code (accessed 21 Jul 2020).

29 Natural England. The People and Nature Survey for England: Monthly interim indicators for April 2020 (Experimental Statistics) . Nat. Engl. 2020.https://www.gov.uk/government/publications/the-people-and-nature-survey-for-england-monthly-interim-indicators-for-april-2020-experimental-statistics/the-people-and-nature-survey-for-england-monthly-interim-indicators-for-april-2020-experimental-statistics (accessed 25 Jun 2020).

30 Natural England. The People and Nature Survey For England. 2020. http://publications.naturalengland.org.uk/publication/6382837173583872

31 Curtis DS, Rigolon A, Schmalz DL, *et al.* Getting out while staying in: Park use decreased during the COVID-19 pandemic, especially where park availability was low. *Cent Open Sci OSF Prepr* Published Online First: 2020. doi:10.31235/osf.io/9xzgf

32 Barton D, Haase D, Mascarenhas A, *et al.* Enabling Access to Greenspace During the Covid-19 Pandemic-Perspectives from Five Cities. Nat. Cities. 2020.https://www.thenatureofcities.com/2020/05/04/enabling-access-to-greenspace-during-the-covid-19-pandemic-perspectives-from-five-cities/ (accessed 23 Oct 2020).

33 Savage M. Lockdown, what lockdown? Sweden's unusual response to coronavirus. BBC News. 2020.

34 Ipsos MORI. Gender differences in attitudes towards Coronavirus. 2020. https://www.ipsos.com/sites/default/files/ct/news/documents/2020-05/fawcett_society_presentation.pdf (accessed 10 Jul 2020).

35 Ipsos MORI, The Fawcett Society. 6 in 10 women finding it harder to stay positive day-to-day due to Coronavirus: Women more likely to bearing the emotional and impact of the pandemic . Fawcett Soc. 2020.https://www.fawcettsociety.org.uk/Handlers/Download.ashx?IDMF=f173fde3-4edb-4c51-8453-7244e867ed90 (accessed 10 Jul 2020).

36 Thompson CW, Aspinall P, Bell S, *et al.* "It Gets You Away From Everyday Life":

For peer review only - http://bmjopen.bmj.com/site/about/guidelines.xhtml

Local Woodlands and Community Use—What Makes a Difference? *Landsc Res* 2007;**30**:109–46. doi:10.1080/0142639042000324794

37 Burgess J. Focusing on Fear: The Use of Focus Groups in a Project for the Community Forest Unit, Countryside Commission. 1996.

38 Richardson EA, Mitchell R. Gender differences in relationships between urban green space and health in the United Kingdom. *Soc Sci Med* 2010;**71**:568–75. doi:10.1016/J.SOCSCIMED.2010.04.015

39 O'Brien L, Morris J. Well-being for all? The social distribution of benefits gained from woodlands and forests in Britain. *Local Environ Int J Justice Sustain* 2014;**19**:356–83. doi:10.1080/13549839.2013.790354

40 Public Health England. Disparities in the risk and outcomes from COVID-19. London: 2020. www.facebook.com/PublicHealthEngland (accessed 26 Jul 2020).

41 Office for National Statistics. Deaths registered weekly in England and Wales, provisional: week ending 10 July 2020. ONS. 2020.https://www.ons.gov.uk/peoplepopulationandcommunity/birthsdeathsandmarriages/deaths/bulletins/deathsregisteredweeklyinenglandandwalesprovisional/latest#deaths-registered-by-age-group (accessed 26 Jul 2020).

42 TNS. Scotland's People and Nature Survey 2013/14. (Scottish Natural Heritage Commissioned Report No. 679). 2014.

43 Johnson B. Prime Minister's statement on coronavirus (COVID-19): 16 March 2020. GOV.UK. 2020.https://www.gov.uk/government/speeches/pm-statement-on-coronavirus-16-march-2020 (accessed 26 Jun 2020).

44 Office for National Statistics. Coronavirus and how people spent their time under lockdown. ONS. 2020.https://www.ons.gov.uk/economy/nationalaccounts/satelliteaccounts/bulletins/coronavirusandhowpeoplespenttheirtimeunderrestrictions/28marchto26april2020 (accessed 16 Jul 2020).

45 Honey-Rosés J, Anguelovski I, Chireh VK, *et al.* The impact of COVID-19 on public space: an early review of the emerging questions – design, perceptions and inequities. *Cities Heal* 2020;:1–17. doi:10.1080/23748834.2020.1780074

46 Moore SA, Faulkner G, Rhodes RE, *et al.* Impact of the COVID-19 virus outbreak on movement and play behaviours of Canadian children and youth: a national survey. *Int J Behav Nutr Phys Act* 2020;**17**. doi:10.1186/s12966-020-00987-8

For peer review only - http://bmjopen.bmj.com/site/about/guidelines.xhtml

Supplementary Materials

Supplementary Table 1: Category mergers of the response categories (ethnicity, age, social grade, sex and dog ownership).

	Original categories (Unweighted N)	New categories (Unweighted N)
**Ethnicity**	White British (1659)	White (1745)
	Any other white background (86)	
	White and Black Caribbean (6)	BAME (87)
	White and Black African (4)	
	White and Asian (8)	
	Any other mixed background (8)	
	Indian (17)	
	Pakistani (5)	
	Bangladeshi (4)	
	Any other Asian background (3)	
	Black Caribbean (4)	
	Black African (3)	
	Any other black background (3)	
	Chinese (10)	
	Other ethnic group (12)	
	Prefer not to say (19)	Missing (420)
	Not answered (401)	
**Age**	18-24 (165)	18-24 (165)
	25-34 (316)	25-64 (1497)
	35-44 (396)	
	45-54 (398)	
	55-64 (387)	
	65+ (590)	65+ (590)
**Social grade**	ABC1 (1419)	
	C2DE (833)	
**Sex**	Male (1018)	
	Female (1234)	
**Dog ownership**	Yes (616)	
	No (1636)	

For peer review only

Supplementary Table 2: Multinomial logistic regression models of being in each outcome group for change in time visiting green space (increase or decrease in visits to green space since movement restrictions were enforced compared to before), results are reported as Risk Ratios (RR) with 95% Lower Confidence Limits (LCL) and Upper Confidence Limits (UCL); **p<0.05**.

		RR of Decreased visits to "Same" and 95% CIs			RR of Increased to "Same" and 95% CIs		
		RR	LCL	UCL	RR	LCL	UCL
	**Intercept**	3.11	2.48	3.90	0.96	0.71	1.30
Sex	**Female *(Ref=Male)***	**1.34**	**1.07**	**1.69**	1.19	0.85	1.66
Social Grade	**C2DE *(Ref=ABC1)***	**0.70**	**0.56**	**0.88**	**0.40**	**0.28**	**0.56**
Age group	**18-24 *(Ref=25-64)***	0.91	0.56	1.47	1.50	0.82	2.73
	**65+ *(Ref=25-64)***	1.13	0.87	1.46	**0.64**	**0.43**	**0.97**
Ethnicity	**BAME *(Ref=White)***	0.62	0.38	1.02	0.83	0.42	1.62
Dog Ownership	**Yes *(Ref=No)***	**0.69**	**0.53**	**0.88**	**0.56**	**0.38**	**0.82**

For peer review only

Supplementary Table 3: Multinomial logistic regression models of being in each outcome group for levels of agreement that green space benefits their mental health more now (since movement restrictions were enforced compared to before), results are reported as Risk Ratios (RR) with 95% Lower Confidence Limits (LCL) and Upper Confidence Limits (UCL); **p<0.05**.

		RR of Agree to "Neither agree or disagree" and 95% CIs			RR of Disagree to "Neither agree or disagree" and 95% CIs		
		RR	LCL	UCL	RR	LCL	UCL
	**Intercept**	2.94	2.17	3.99	0.43	0.27	0.70
Sex	**Female *(Ref=Male)***	**1.72**	**1.24**	**2.39**	1.29	0.76	2.18
Social Grade	**C2DE *(Ref=ABC1)***	**0.67**	**0.48**	**0.93**	0.88	0.52	1.49
Age group	**18-24 *(Ref=25-64)***	0.87	0.40	1.89	**2.71**	**1.03**	**7.12**
	**65+ *(Ref=25-64)***	**0.47**	**0.33**	**0.67**	**0.37**	**0.19**	**0.73**
Ethnicity	**BAME *(Ref=White)***	1.20	0.54	2.69	0.21	0.03	1.43
Dog Ownership	**Yes *(Ref=No)***	0.72	0.51	1.02	1.02	0.59	1.76

For peer review only

Supplementary Table 4: Multinomial logistic regression models of being in each outcome group for levels of agreement that respondents missed seeing/talking to people in green space more since movement restrictions were enforced compared to before, results are reported as Risk Ratios (RR) with 95% Lower Confidence Limits (LCL) and Upper Confidence Limits (UCL); **p<0.05**.

		RR of Agree to "Neither agree or disagree" and 95% CIs			RR of Disagree to "Neither agree or disagree" and 95% CIs		
		RR	LCL	UCL	RR	LCL	UCL
	**Intercept**	1.47	1.08	1.99	0.89	0.62	1.27
Sex	**Female *(Ref=Male)***	**1.53**	**1.11**	**2.13**	0.76	0.50	1.14
Social Grade	**C2DE *(Ref=ABC1)***	0.76	0.54	1.06	0.90	0.60	1.36
Age group	**18-24 *(Ref=25-64)***	1.40	0.69	2.86	1.00	0.40	2.49
	**65+ *(Ref=25-64)***	0.98	0.67	1.43	0.78	0.49	1.26
Ethnicity	**BAME *(Ref=White)***	0.91	0.43	1.93	0.72	0.27	1.91
Dog Ownership	**Yes *(Ref=No)***	1.33	0.92	1.91	1.15	0.74	1.80

For peer review only

Supplementary Table 5: Multinomial logistic regression models of being in each outcome group for levels of agreement that respondents do more physical activity in green space following the movement restrictions, results are reported as Risk Ratios (RR) with 95% Lower Confidence Limits (LCL) and Upper Confidence Limits (UCL); **p<0.05**.

		RR of Agree to "Neither agree or disagree" and 95% CIs			RR of Disagree to "Neither agree or disagree" and 95% CIs		
		RR	LCL	UCL	RR	LCL	UCL
	**Intercept**	1.09	0.78	1.51	1.22	0.89	1.66
Sex	**Female *(Ref=Male)***	1.44	0.99	2.08	1.35	0.97	1.88
Social Grade	**C2DE *(Ref=ABC1)***	0.75	0.51	1.09	0.85	0.61	1.19
Age group	**18-24 *(Ref=25-64)***	2.07	0.99	4.36	1.13	0.52	2.49
	**65+ *(Ref=25-64)***	**0.51**	**0.32**	**0.80**	0.93	0.64	1.34
Ethnicity	**BAME *(Ref=White)***	0.95	0.44	2.08	**0.38**	**0.15**	**0.97**
Dog Ownership	**Yes *(Ref=No)***	**0.43**	**0.28**	**0.66**	0.95	0.68	1.35

For peer review only

Supplementary Table 6: Change in green space visit frequency since restrictions by individual demographic and socio-economic characteristics (unweighted).

	Visited green space in the year before restrictions		Visited green space after restrictions (only those who visited green space before restrictions)		Green space visitation change since lockdown		
	**Yes (N=2045, 93.5%)**	**No (N=142, 6.4%)**	**Yes (N=1099, 53.8%)**	**No (N=943, 46.2%)**	**Decreased (N=1392, 63.3%)**	**Same (N=473, 21.5%)**	**Increased (N=333, 15.2%)**
	% *(N)*	% *(N)*	% *(N)*	% *(N)*	% *(N)*	% *(N)*	% *(N)*
Sex							
**Male (ref)**	92.6% *(919)*	7.4% *(73)*	55.2% *(507)*	44.8% *(411)*	61.6% *(609)*	23.5% *(232)*	15% *(148)*
**Female**	94.2% *(1126)*	5.8% *(69)*	52.7% *(592)*	47.3% *(532)*	64.8% *(783)*	19.9% *(241)*	15.3% *(185)*
Social grade							
**ABC1 (ref)**	95.8% *(1328)*	4.2% *(58)*	56.7% *(753)*	43.3% *(574)*	62.9% *(871)*	18.8% *(260)*	18.3% *(254)*
**C2DE**	89.5% *(717)*	10.5% *(84)*	48.4% *(346)*	51.6% *(369)*	64.1% *(521)*	26.2% *(213)*	9.7% *(79)*
Age							
**18-24**	93.3% *(139)*	6.7% *(10)*	56.9% *(78)*	43.1% *(59)*	58.4% *(87)*	20.8% *(31)*	20.8% *(31)*
**25-64 (ref)**	93.6% *(1364)*	6.4% *(94)*	56.1% *(765)*	43.9% *(598)*	61.8% *(906)*	21.5% *(315)*	16.7% *(245)*
**65+**	93.4% *(542)*	6.6% *(38)*	47.2% *(256)*	52.8% *(286)*	68.4% *(399)*	21.8% *(127)*	9.8% *(57)*
Ethnicity							
**White (ref)**	93.6% *(1592)*	6.4% *(109)*	52.1% *(828)*	47.9% *(762)*	64.1% *(1098)*	22.5% *(385)*	13.4% *(229)*
**BAME**	84.1% *(69)*	15.9% *(13)*	47.8% *(33)*	52.2% *(36)*	57% *(45)*	30.4% *(24)*	12.7% *(10)*
Dog ownership							
**Don't own a dog/s (ref)**	93.1% *(1476)*	6.9% *(109)*	51.4% *(757)*	48.6% *(717)*	64.4% *(1027)*	19.5% *(311)*	16.1% *(256)*
**Own a dog/s**	94.5% *(569)*	5.5% *(33)*	60.2% *(342)*	39.8% *(226)*	60.4% *(365)*	26.8% *(162)*	12.7% *(77)*

Don't know/ can't recall responses were all excluded from analysis.

For peer review only

Supplementary Table 7: Change in green space experience since restrictions by individual demographic and socio-economic characteristics (unweighted).

	**Using green spaces benefits my mental health more now**			**Miss social interaction in green spaces now**			**Do more physical activity in green spaces now**		
	**Agree (N=721, 65.8%)**	**Neither (N=272, 24.8%)**	**Disagree (N=102, 9.3%)**	**Agree (N=590, 54.1%)**	**Neither (N=288, 26.4%)**	**Disagree (N=213, 19.5%)**	**Agree (N=322, 29.5%)**	**Neither (N=337, 30.9%)**	**Disagree(N=431, 39.5%)**
	% *(N)*		% *(N)*	% *(N)*		% *(N)*	% *(N)*		% *(N)*
Sex									
**Male (ref)**	59.6% *(300)*	30.8% *(155)*	9.5% *(48)*	45.8% *(231)*	30% *(151)*	24.2% *(122)*	27.4% *(137)*	35.4% *(177)*	37.2% *(186)*
**Female**	71.1% *(421)*	19.8% *(117)*	9.1% *(54)*	61.2% *(359)*	23.3% *(137)*	15.5% *(91)*	31.4% *(185)*	27.1% *(160)*	41.5% *(245)*
Social grade									
**ABC1 (ref)**	68% *(511)*	22.9% *(172)*	9.1% *(68)*	54.9% *(412)*	25.3% *(190)*	19.7% *(148)*	31.6% *(236)*	28.9% *(216)*	39.6% *(296)*
**C2DE**	61% *(210)*	29.1% *(100)*	9.9% *(34)*	52.2% *(178)*	28.7% *(98)*	19.1% *(65)*	25.1% *(86)*	35.4% *(121)*	39.5% *(135)*
Age									
**18-24**	67.5% *(52)*	19.5% *(15)*	13% *(10)*	64.9% *(50)*	22.1% *(17)*	13% *(10)*	48.7% *(38)*	16.7% *(13)*	34.6% *(27)*
**25-64 (ref)**	69% *(526)*	21% *(160)*	10% *(76)*	53.7% *(407)*	25.9% *(196)*	20.4% *(155)*	30.8% *(233)*	30.1% *(228)*	39.1% *(296)*
**65+**	55.9% *(143)*	37.9% *(97)*	6.2% *(16)*	52% *(133)*	29.3% *(75)*	18.8% *(48)*	20% *(51)*	37.6% *(96)*	42.4% *(108)*
Ethnicity									
**White (ref)**	62.8% *(518)*	27.3% *(225)*	9.9% *(82)*	51.6% *(423)*	28% *(230)*	20.4% *(167)*	26.5% *(218)*	32.3% *(266)*	41.2% *(339)*
**BAME**	75% *(24)*	21.9% *(7)*	3.1% *(1)*	51.5% *(17)*	30.3% *(10)*	18.2% *(6)*	38.7% *(12)*	38.7% *(12)*	22.6% *(7)*
Dog ownership									
**Don't own a dog/s (ref)**	68.2% *(514)*	23.9% *(180)*	8% *(60)*	52.5% *(395)*	27.1% *(204)*	20.5% *(154)*	34.5% *(259)*	28.7% *(215)*	36.8% *(276)*
**Own a dog/s**	60.7% *(207)*	27% *(92)*	12.3% *(42)*	57.7% *(195)*	24.9% *(84)*	17.5% *(59)*	18.5% *(63)*	35.9% *(122)*	45.6% *(155)*

Don't know/ can't recall responses were all excluded from analysis.

For peer review only

STROBE Statement—Checklist of items that should be included in reports of ***cross-sectional studies***

	Item No	Recommendation	Page No
**Title and abstract**	1	(*a*) Indicate the study's design with a commonly used term in the title or the abstract	P1
		(*b*) Provide in the abstract an informative and balanced summary of what was done and what was found	P2
**Introduction**			
Background/rationale	2	Explain the scientific background and rationale for the investigation being reported	P3/4
Objectives	3	State specific objectives, including any prespecified hypotheses	P3/4
**Methods**			
Study design	4	Present key elements of study design early in the paper	P4/5
Setting	5	Describe the setting, locations, and relevant dates, including periods of recruitment, exposure, follow-up, and data collection	P4/5
Participants	6	(*a*) Give the eligibility criteria, and the sources and methods of selection of participants	P4/5
Variables	7	Clearly define all outcomes, exposures, predictors, potential confounders, and effect modifiers. Give diagnostic criteria, if applicable	P4-6
Data sources/ measurement	8*	For each variable of interest, give sources of data and details of methods of assessment (measurement). Describe comparability of assessment methods if there is more than one group	P4-6
Bias	9	Describe any efforts to address potential sources of bias	P4, 6
Study size	10	Explain how the study size was arrived at	P4
Quantitative variables	11	Explain how quantitative variables were handled in the analyses. If applicable, describe which groupings were chosen and why	P5-7
Statistical methods	12	(*a*) Describe all statistical methods, including those used to control for confounding	P6/7
		(*b*) Describe any methods used to examine subgroups and interactions	-
		(*c*) Explain how missing data were addressed	P4-7
		(*d*) If applicable, describe analytical methods taking account of sampling strategy	P4-6
		(*e*) Describe any sensitivity analyses	-
**Results**			
Participants	13*	(a) Report numbers of individuals at each stage of study—eg numbers potentially eligible, examined for eligibility, confirmed eligible, included in the study, completing follow-up, and analysed	Supplementary Tables 1, 6 and 7
		(b) Give reasons for non-participation at each stage	-
		(c) Consider use of a flow diagram	-

For peer review only - http://bmjopen.bmj.com/site/about/guidelines.xhtml

Descriptive data	14*	(a) Give characteristics of study participants (eg demographic, clinical, social) and information on exposures and potential confounders	P4/5, Supplementary Table 1
		(b) Indicate number of participants with missing data for each variable of interest	Table 1 + Supplementary Table 1
Outcome data	15*	Report numbers of outcome events or summary measures	Tables 2-4+ Supplementary Tables 2-7
Main results	16	(*a*) Give unadjusted estimates and, if applicable, confounder-adjusted estimates and their precision (eg, 95% confidence interval). Make clear which confounders were adjusted for and why they were included	P7-13
		(*b*) Report category boundaries when continuous variables were categorized	-
		(*c*) If relevant, consider translating estimates of relative risk into absolute risk for a meaningful time period	-
Other analyses	17	Report other analyses done—eg analyses of subgroups and interactions, and sensitivity analyses	-
**Discussion**			
Key results	18	Summarise key results with reference to study objectives	P13/14
Limitations	19	Discuss limitations of the study, taking into account sources of potential bias or imprecision. Discuss both direction and magnitude of any potential bias	P15/16
Interpretation	20	Give a cautious overall interpretation of results considering objectives, limitations, multiplicity of analyses, results from similar studies, and other relevant evidence	P13-15
Generalisability	21	Discuss the generalisability (external validity) of the study results	P13, 15/16
**Other information**			
Funding	22	Give the source of funding and the role of the funders for the present study and, if applicable, for the original study on which the present article is based	P17

*Give information separately for exposed and unexposed groups.

Note: An Explanation and Elaboration article discusses each checklist item and gives methodological background and published examples of transparent reporting. The STROBE checklist is best used in conjunction with this article (freely available on the Web sites of PLoS Medicine at http://www.plosmedicine.org/, Annals of Internal Medicine at http://www.annals.org/, and Epidemiology at http://www.epidem.com/). Information on the STROBE Initiative is available at www.strobe-statement.org.

For peer review only
